# Insulin signaling in *Drosophila melanogaster* mediates Aβ toxicity

**DOI:** 10.1038/s42003-018-0253-x

**Published:** 2019-01-08

**Authors:** Yunpeng Huang, Zhihui Wan, Zhiqing Wang, Bing Zhou

**Affiliations:** 1 State Key Laboratory of Membrane Biology, School of Life Sciences, Tsinghua University, 100084 Beijing, China; 2 Beijing Institute for Brain Disorders, 45 Changchun St, 100053 Beijing, China

## Abstract

Alzheimer’s disease (AD) and diabetes are clinically positively correlated. However, the connection between them is not clarified. Here, using *Drosophila* as a model system, we show that reducing insulin signaling can effectively suppress the toxicity from Aβ (Amyloid beta 42) expression. On the other hand, Aβ accumulation led to the elevation of fly insulin-like peptides (ILPs) and activation of insulin signaling in the brain. Mechanistically, these observations are attributed to a reciprocal competition between *Drosophila* insulin-like peptides and Aβ for the activity of insulin-degrading enzyme (IDE). Intriguingly, peripheral insulin signaling is decreased despite its heightened activity in the brain. While many upstream factors may modify Aβ toxicity, our results suggest that insulin signaling is the main downstream executor of Aβ damage, and thus may serve as a promising target for Alzheimer’s treatment in non-diabetes patients. This study explains why more Alzheimer’s cases are found in diabetes patients.

## Abstract (web summary)

Huang et al. show in Drosophila that amyloid beta (Aβ) toxicity is mediated by increased insulin signaling. A competition between insulin-like peptides and Aβ for the activity of insulin-degrading enzyme may explain why many Alzheimer’s disease patients also suffer from diabetes.

## Introduction

Alzheimer’s disease (AD) is the most common type of neurodegenerative disease among the elderly, leading to decline of memory, behavioral performance, and cognition. The extracellular senile plaque, an aggregation of β-amyloid peptide (Aβ), and intracellular neurofibrillary tangles (NFTs), comprised of microtubule-associated protein Tau, are considered the two major hallmarks of this progressive neuropathology. Among factors that may affect late-onset AD, age is the major risk factor. AD incidence increases to 15% among people aged over 65 and further increases to 40–50% among people aged over 85^1–4^. At present, it is not clear how aging may increase the risk of AD and which factors of the aging process are involved in the progress and pathology of AD.

Diabetes is also a risk factor for AD and dementia^5–7^. In particular, type 2 diabetes increases the risk of developing cognitive impairment and dementia^8,9^. Type 2 diabetes and AD share several common symptoms such as hyperglycemia, hyperinsulinemia, and insulin resistance. Type 2 diabetes may facilitate the progress of AD, and AD increases the risk of developing type 2 diabetes^10,11^. Obviously, there is an intimate link between AD and type 2 diabetes, but it is not clear how they are mechanistically linked. Although AD patients tend to be physiologically overall insulin resistant, evidence also suggests that the level of insulin/insulin signaling is unchanged or increased in AD brains^12^, and downstream activity like AKT (also known as PKB, protein kinase B) is upregulated^13,14^. Consistently, insulin level in the serum of AD patients is higher than in healthy subjects^15^, and hyperinsulinemia is associated with increased risk of AD^16^. Generally speaking, the current literature is very confusing with regard to how AD and insulin are connected. It has been shown that insulin treatment can modestly improve cognition, but also worsen AD pathology^10,17–19^, whereas decreasing insulin signaling can aggravate or protect from AD pathology^20^. The relationship between insulin signaling and AD thus starts to get bewildering. If type 2 diabetes is associated with AD, then reduced insulin signaling might underlie AD, but why would hyperinsulinemia or insulin treatment of type 2 diabetes aggravate AD?

In the course of studying how ageing relates to AD in *Drosophila*, we found reduction of insulin signaling efficiently ameliorates Aβ toxicity. Downregulation of *Drosophila* insulin-like peptides (ILPs), which are secreted by neurons in the brain^21^, and multiple other insulin signaling components, could drastically suppress Aβ toxicity. As insulin signaling has been established as an important player in ageing, our findings link ageing, AD, and type 2 diabetes, and indicate insulin signaling is the common underpinning of these biological phenomena. We speculate that in some type 2 diabetes patients insulin insensitivity may not uniformly occur in all tissues. It is therefore possible that compensatory effects due to type 2 diabetes may hyperactivate insulin signaling in certain brain regions where insulin signaling was originally less affected, thus aggravating AD.

## Results

### *chico* downregulation mitigates Aβ toxicity

Since ageing is the single most important contributing factor to AD, and ageing and neurodegeneration share common features, we explored whether ageing factors underlie AD. A group of upstream activation sequence (UAS)-driven RNA interference (RNAi) transgenes targeting genes known to be involved in ageing (Supplementary Table 1) were crossed into the AD model fly *Elav-Gal4; UAS-Aβ42*, which expresses Aβ42 in the central nervous system (CNS) (Elav-Gal4 specifically directs UAS-fused gene expression in the CNS, Supplementary Fig. 1a)^22^. Expression of Aβ42 in the fly CNS causes neurodegeneration and brain vacuolization, resulting in severe climbing disability and lifespan reduction^22^. For technical reasons, we mainly examined locomotion loss and degree of vacuolization as indicators of Aβ42 toxicity.

**Fig. 1:**
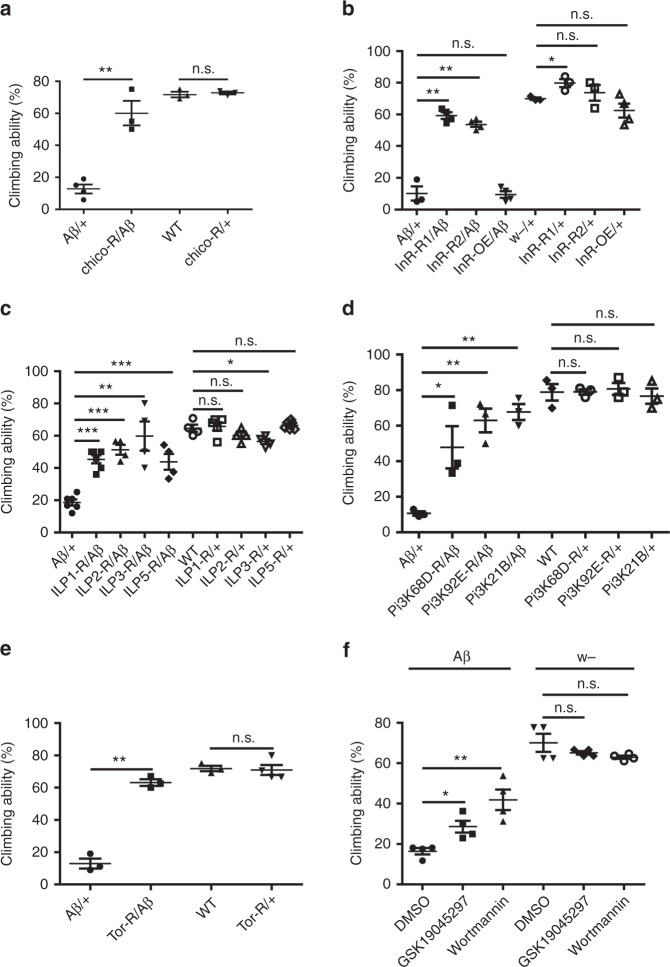
Genetic screening identifies insulin signaling as a significant modifier of Aβ toxicity. **a** Effects of *chico* RNAi on the climbing ability of Aβ flies. *Elav-Gal4* was used to drive the expression of Aβ and the knock-down of *chico*. Data represent mean ± SEM, **p < 0.01. *Elav-Gal4* was used to express Aβ and knock-down ILPs in fly CNS. The corresponding genotypes: “Aβ/+” is *Elav-Gal4*>*UAS-Aβ*/+; “*chico*-R/Aβ” is *Elav-Gal4*>*UAS-Aβ*/*UAS-chico RNAi*; “WT” is *Elav-Gal4*>+/+; “*chico*-R/+” is *Elav-Gal4*>*UAS-chico RNAi*/+. **b** Effects of *InR* manipulation on the climbing ability of Aβ flies. *Elav-Gal4* was used to drive the overexpression of *InR*, Aβ and the knock-down of *InR*. Data represent mean ± SEM, *p < 0.05, **p < 0.01. The corresponding genotypes: “Aβ/+” is *Elav-Gal4*>*UAS-Aβ*/+; “*InR*-R/Aβ” is *Elav-Gal4*>*UAS-Aβ*/*UAS-InR RNAi*; “InR-OE/Aβ” is *Elav-Gal4*>UAS-*InR*/Aβ; “w^−^/+” is *Elav-Gal4*>*w*^−^/+; “*InR*-R/+” is Elav-Gal4>UAS-*InR* RNAi/+; “InR-OE/+” is *Elav-Gal4*>*UAS-InR*/+; *w*^−^ was the background fly for *InR* RNAi and overexpression (OE) transgenes. R RNAi, OE overexpression. **c** Downregulation of fly ILPs improves the climbing ability of Aβ flies. *Elav-Gal4* was used to express Aβ and knock-down ILPs in fly CNS. Data represent mean ± SEM, *p < 0.05, **p < 0.01, ***p < 0.001. **d** Effects of fly *Pi3K68D* and *Pi3K21B* RNAi on the climbing ability of Aβ flies. *Elav-Gal4* was used to express Aβ and knock-down *Pi3K* in fly CNS. Data represent mean ± SEM, *p < 0.05, **p < 0.01. **e** Effects of fly *Tor* RNAi on the climbing ability of Aβ flies. *Elav-Gal4* was used to express Aβ and knock-down *Tor* in fly CNS. Data represent mean ± SEM, **p < 0.01. **f** Effects of inhibitors on the climbing ability of Aβ flies. InR inhibitor GSK19045297 and PI3K inhibitor Wortmannin could improve the mobility of Aβ flies. *Elav-Gal4* was used to express Aβ in fly CNS. Data represent mean ± SEM, *p < 0.05, **p < 0.01. *Elav-Gal4* was used to drive Aβ expression in fly CNS

**Fig. 2:**
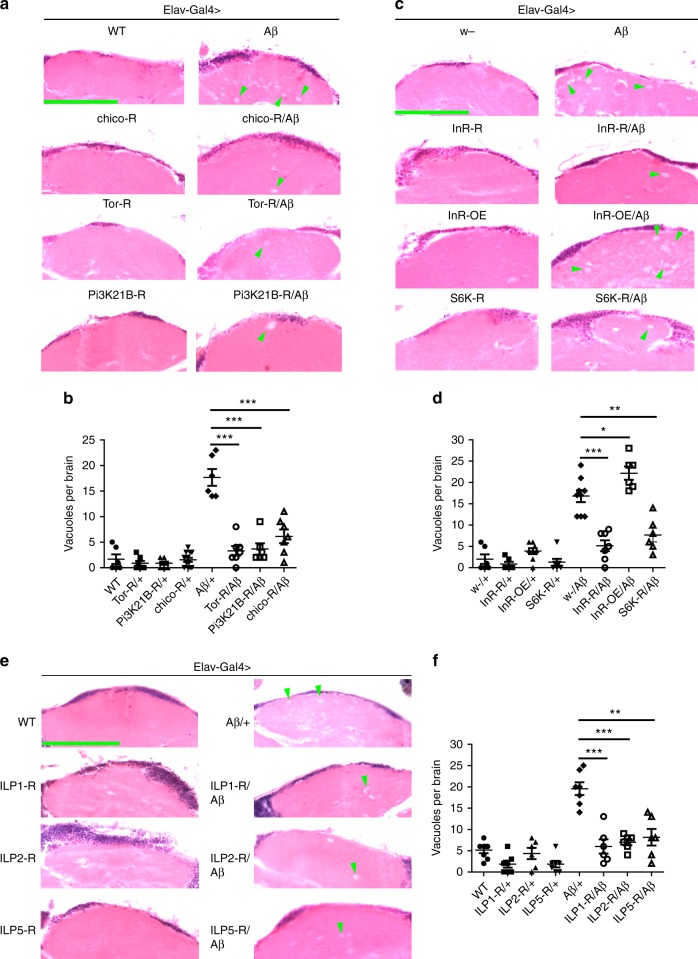
Downregulating insulin signaling reduces neurodegeneration in Aβ flies. **a**–**d** Modulations of fly insulin signaling by RNAi or overexpression of the downstream components can effectively affect the number of vacuoles in the brains of Aβ flies. *Elav-Gal4* was used to overexpress and knock-down the corresponding genes. **a**, **c** Paraffin brain sections were stained with H&E. Green arrow heads indicate the degenerative vacuoles in flies’ brains. Scale bar: 50 μm. **b** Quantification of (**a**). Data represent mean ± SEM, *p < 0.05, **p < 0.01, ***p < 0.001. **d** The quantification of (**c**). Data represent mean ± SEM, ***p < 0.001. **e**, **f** H&E-stained paraffin brain sections of the Aβ, and Aβ, ILPs RNAi flies. *Elav-Gal4* was used to express Aβ and knock-down the corresponding genes. Green arrow heads indicate the degenerative vacuoles in flies’ brains. Scale bar: 50 μm. **f** Quantification of (**e**). Data represent mean ± SEM, **p < 0.01, ***p < 0.001

We found downregulation of fly ageing genes *chico*, *Indy* (I’m not dead yet) and *mth* (*methuselah*) significantly improved the climbing ability of AD flies (Supplementary Fig. 1b), indicating downregulation of these genes could modify Aβ toxicity. As *chico* is the homolog of mammalian insulin receptor substrate (IRS), and the only one with a clearly characterized pathway, we decided to further analyze this gene and its likely pathway. Knocking-down *chico* improved the climbing ability of Aβ flies, from 16% to 60% without any adverse effect on the climbing ability of wild-type (WT) flies (Fig. 1a). RNAi efficacy was confirmed by reverse transcription-PCR (RT-PCR) (Supplementary Fig. 2). Expression of human Aβ42 in the fly CNS driven by *Elav-Gal4* causes severe neurodegeneration including brain vacuolization^22,23^. In order to evaluate neurodegeneration rescue by *chico* knock-down, sections of fly heads were stained with hematoxylin–eosin (H&E staining) and examined under the microscope for degenerative vacuolization. We found that *chico* RNAi dramatically decreased the number of vacuoles in Aβ fly brains (Fig. 2a, b), indicating it could suppress neurodegeneration of the Aβ fly.

### Insulin signaling pathway mediates Aβ toxicity

To ascertain whether other components in the insulin pathway besides *chico* are involved in AD, we first tested the effect of RNAi and overexpression of *InR* (insulin receptor) (Supplementary Fig. 2). Knocking-down *InR* in Aβ flies dramatically improved the climbing ability of Aβ flies (Fig. 1b); two independent lines improved the climbing ability of Aβ flies from 15% to 60% and 58%, respectively (Fig. 1b). As a control, the effects of *InR* RNAi on normal flies were tested. One *InR* RNAi line (line 2#) did not affect the climbing ability of normal flies, whereas the other RNAi line (line 1#) slightly improved the climbing ability of WT flies (Fig. 1b), probably as a result of *InR*’s effects on aging and related climbing disability. However, overexpression of *InR* in Aβ flies did not significantly worsen the climbing disability of Aβ flies (Fig. 1b), likely because the climbing disability was already very severe in the aged Aβ flies (~30 days old flies). Consistently, *Drosophila* ILPs knock-down also suppressed Aβ toxicity. It has been reported that ILP1, 2, 3, and 5 are all expressed in the fly brain^24^. Individual knock-down of *Drosophila ILP1*, *ILP2*, and *ILP5* by using Elav-Gal4 (Elav-Gal4 directs expression in the brain including the insulin-producing cells (IPCs), Supplementary Fig. 1c), could effectively rescue the climbing disability of Aβ flies (Fig. 1c); the climbing ability was restored from 20% to ~40–50% of the normal, whereas knocking-down *ILP1*, *ILP2*, and *ILP5* did not affect the climbing ability of the control flies (Fig. 1c). *ILP3* RNAi, however, had some side effects on its own (causing the impairment of the climbing ability in the control flies), making the analysis difficult. We therefore did not pursue ILP3 further.

We then analyzed some downstream components of insulin signaling such as phosphatidylinositol-4,5-bisphosphate 3-kinase (Pi3K) and target of rapamycin (Tor)^25^ for their Aβ-toxicity-rescuing effects. Knocking-down Pi3K components *Pi3K68D* and *Pi3K21B* could both improve the climbing ability of Aβ flies, whereas their effects on the WT flies were not significant (Fig. 1d). *Tor* downregulation had similar climbing ability-rescuing effects (Fig. 1e).

We additionally used Cha-Gal4 (expressed in fly cholinergic neurons) to confirm the results. Knocking-down of *InR* and *chico* by Cha-Gal4 could also mitigate the climbing disability of the Aβ expression fly (Supplementary Fig. 3a).

To exclude the possibility that the rescue effect was a secondary effect of early stage gene expression, we adopted the RU486-induced gene switch system to examine insulin signaling effects on Aβ toxicity. With this drug-induced expression system, we could analyze the effect of genes specifically on adult stages, bypassing the early development period. Knocking-down insulin signaling components in the adult stage could still rescue the climbing disability caused by Aβ expression, as *InR*, *chico*, *Pi3K21B*, and *ILP1/2* RNAi all could suppress Aβ toxicity (Supplementary Fig. 3b and Supplementary Fig. 3c).

In addition to genetic interference, we further used chemical drugs to examine the effects of insulin signaling on Aβ toxicity. Inhibitors of both InR (GSK19045297) and Pi3K (Wortmannin) significantly improved the climbing ability of Aβ flies (Fig. 1f), confirming that inhibition of insulin signaling can rescue Aβ toxicity.

The degenerative process in the brain of Aβ flies was further analyzed. RNAi of *InR*, *Pi3K21B*, and *Tor* reduced the number of brain vacuoles in Aβ flies (Fig. 2a–d). H&E staining of brain sections revealed similar rescuing effects for ILPs RNAi: the number of brain vacuoles in Aβ flies was significantly reduced when *ILP1*, *ILP2*, and *ILP5* were knocked-down (Fig. 2e, f), indicating insulin signaling, i.e., insulin receptor and its downstream components, mediates the toxicity of Aβ in the fly’s brain.

The above results obtained with both genetic techniques and chemical drugs proved that insulin signaling mediates Aβ toxicity; reduction of insulin signaling suppresses, instead of enhancing, Aβ fly phenotypes.

### Insulin signaling is mis-regulated in Aβ flies

**Fig. 3:**
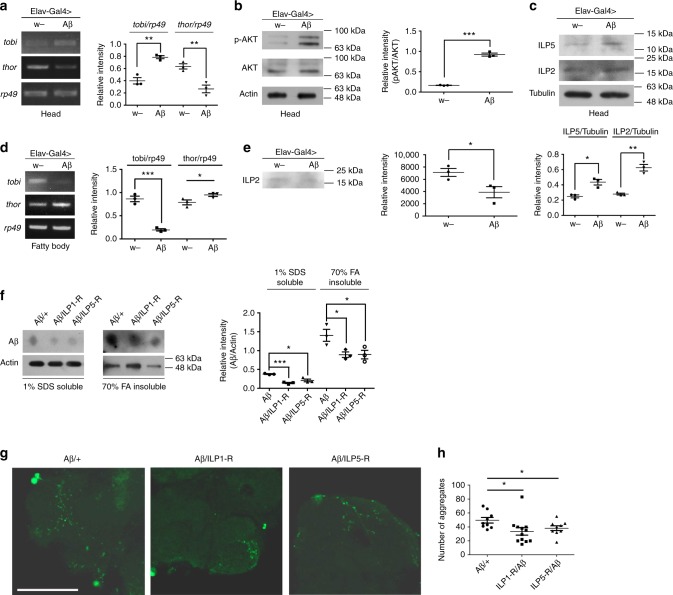
Insulin signaling is mis-regulated in Aβ flies. Elav-Gal4 was used to express Aβ in fly CNS. All results (from **a** to **f**) are reproducible in three independent RT-PCR experiments, and only one representative is shown here. **a** The mRNA levels of target of brain insulin (*tobi*) and fly 4EBP *(thor)* in Aβ flies were analyzed by RT-PCR. *rp49* was used as the loading control. **p < 0.01. **b** The levels of p-AKT (phosphorylated AKT) in Aβ flies were analyzed by western blotting. Total levels of AKT were similar in the control fly and the Aβ fly. Actin was used as the loading control (uncropped images of the blots are presented in Supplementary Fig. 11a). ***p < 0.001. **c** Protein levels of fly insulin-like peptide 5 (ILP5) and insulin-like peptide 2 (ILP2) in control and Aβ flies were analyzed by western blotting. Tubulin was used as the loading control (uncropped images of the blots are presented in Supplementary Fig. 11b). *p < 0.05, **p < 0.01. **d** mRNA levels of *tobi* and fly *thor* in the fat body of Aβ flies were analyzed by RT-PCR. *rp49* was used as the loading control. ***p < 0.001. **e** Protein level of fly ILP2 in the hemolymph of control and Aβ flies was analyzed by western blotting (uncropped image of the blot is presented in Supplementary Fig. 11c). Concentration of total proteins was normalized by BCA method. *p < 0.05. **f**–**h** Downregulation of ILPs effectively inhibits the accumulation of Aβ in fly brains. **f** The levels of soluble and insoluble Aβ in fly heads were analyzed by dot blotting. Actin was used as the loading control (uncropped images of the blots are presented in Supplementary Fig. 11d). 1% SDS fraction represents the level of soluble Aβ; 70% FA fraction represents the level of insoluble Aβ. *p < 0.05. **g**, **h** Immunostaining of Aβ aggregates in fly brains. *Elav-Gal4* was used to express Aβ and knock-down ILPs in fly CNS. Scale bar = 100 μm. **h** The quantification of **g**, *n* = 6. Data represent mean ± SEM, *p < 0.05

As Aβ toxicity is related to the activity of insulin signaling, we asked whether Aβ expression could alter the level of insulin and insulin signaling. In the literature, changes of insulin level and insulin signaling in AD brains are still debated and inconsistent^26^. By using RT-PCR analyses, we found that the head level of *thor (*4EBP homolog in the fly*)*, which is reduced by insulin^27^, was decreased in Aβ flies (Fig. 3a); the level of head *tobi* (target of brain insulin) was increased in Aβ flies (Fig. 3a). These results suggest Aβ expression indeed positively affects InR signaling pathway in the head. To confirm this, we further analyzed the levels of phosphorylated AKT (p-AKT), whose phosphorylation state is regulated in InR signaling^28^. Our results showed that the downstream component AKT was activated in the head of Aβ flies, as revealed by elevated p-AKT (Fig. 3b).

To test whether the effect of Aβ on insulin signaling relies on upstream activation by ligands, we examined the levels of ILP2 and ILP5, two of the major ILPs in the brain^29^. The protein levels of ILP2 and ILP5 were indeed elevated in Aβ flies when compared with those in control flies (Fig. 3c), indicating that Aβ expression led to an increase of ILP2 and ILP5. Immunostaining of Repo and ILP2 indicated that the glial cells and IPCs were not significantly affected in AD flies (Supplementary Fig. 4a and Supplementary Fig. 4b), but ILP2 appeared to be more accumulated in IPCs (Supplementary Fig. 4c).

The level of insulin signaling in peripheral tissues of AD flies was then assayed. *tobi* mRNA level was significantly reduced in the fat body of AD flies (Fig. 3d), but that of *thor* increased, indicating a reduction of insulin signaling in peripheral tissues. This reduction is in contrast to the increase in the brain. Western blot indicated that circulating ILP2 protein signals, albeit very weak, appeared reduced in the hemolymph of Aβ flies (Fig. 3e). We, however, cannot be certain that this is also the case for the other ILPs in the body, given undetectable level of ILP5 and lack of appropriate antibodies to the other ILPs.

We next tested whether ILPs suppression could reciprocally reduce Aβ levels. We separated the soluble and insoluble fractions of Aβ from fly heads and tested by dot blot their individual levels. Both fractions of Aβ were significantly reduced when *ILP1* and *ILP5* were knocked-down (Fig. 3f). Results from Aβ immunostaining also confirmed this observation (Fig. 3g, h). We conclude that ILPs reduction effectively downregulates Aβ toxicity and decreases Aβ level.

### Aβ competes with *Drosophila* ILPs for IDE degradation

**Fig. 4:**
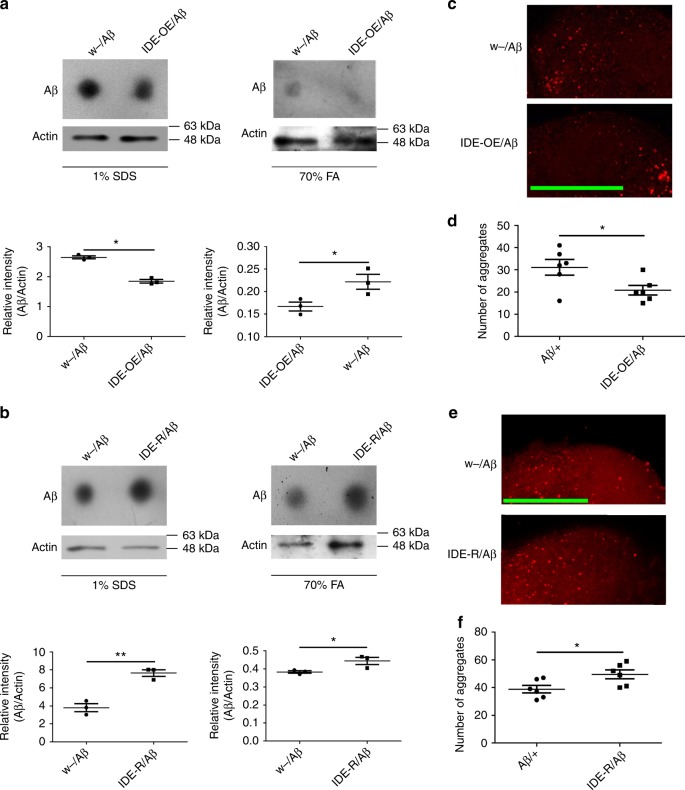
IDE regulates the level of Aβ in vivo. **a**–**d** Manipulating the level of IDE could affect the level of Aβ in fly. *Elav-Gal4* was used to drive the overexpression or knock-down of *IDE*. **a**, **b** The levels of Aβ in *IDE* OE flies and *IDE* RNAi flies were analyzed by dot blotting. Actin was used as the loading control (uncropped images of the blots are presented in Supplementary Fig. 11e and Supplementary Fig. 11f). *Elav-Gal4* was used to drive the overexpression or knock-down of *IDE*. Results are reproducible in three independent dot blotting experiments, and only one is shown here. **c**–**f** The levels of Aβ aggregates were analyzed with immunostaining. (**c**, **d**) The level of Aβ aggregates in *IDE*-overexpression flies, *n* = 6. Scale bar = 50 μm. **d** The quantification of (**c**). Data represent mean ± SEM, *p < 0.05. **e**, **f** The level of Aβ aggregates in *IDE* RNAi flies, *n* = 6. Scale bar = 50 μm. **f** The quantification of (**e**). Data represent mean ± SEM, *p < 0.05

To explore the mechanism of reciprocal regulation between ILPs and Aβ, we focused our attention on a metalloprotease named insulin-degrading enzyme (IDE), which might regulate the level of both Aβ and mammalian insulin^30–34^. Although it has been reported that IDE regulates the level of fly ILPs in vivo^35^, we did not know whether *Drosophila* IDE could regulate the level of Aβ, and if so whether competition between insulin and Aβ for IDE contributed to Aβ toxicity in vivo. To ascertain this, we separated the soluble and insoluble Aβ fractions from the heads of Aβ flies, and tested the level of Aβ with an antibody (Fig. 4a, b). IDE is broadly expressed in the adult fly brain, including the neurons of the mushroom body (Supplementary Fig. 5a). We modulated IDE expression by using Elav-Gal4 (Supplementary Fig. 5b). Overexpression of *Drosophila IDE* (Supplementary Fig. 5b) significantly reduced both the soluble and insoluble Aβ levels, indicating that the total levels of Aβ were reduced in *IDE*-overexpressing flies (Fig. 4a). We also tested Aβ deposition in fly brains. Immunostaining showed that Aβ deposition was reduced when *IDE* was overexpressed (Fig. 4c, d). Correspondingly, RNAi of *IDE* (Supplementary Fig. 5b) led to an increase of Aβ deposition (Fig. 4b, e, f). These results indicate that the level of Aβ in fly brains is regulated by *Drosophila* IDE.

**Fig. 5:**
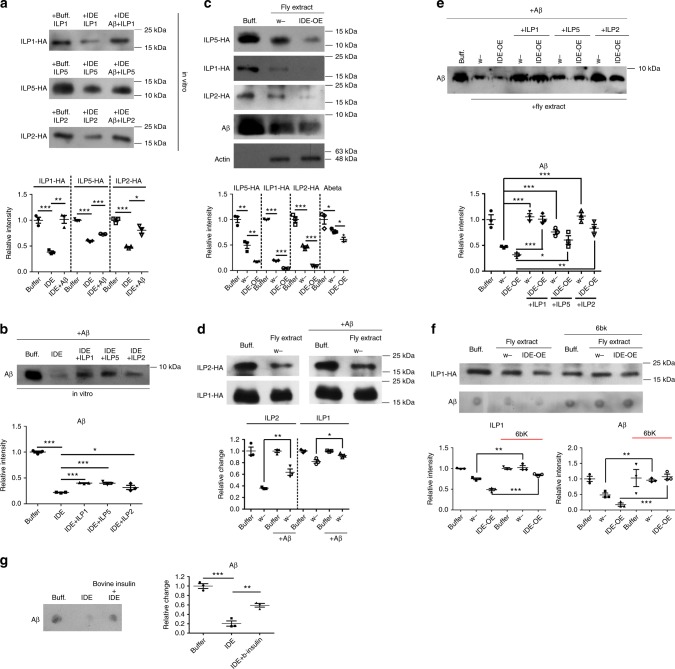
Aβ and ILPs compete for the activity of IDE. **a** Aβ inhibits the ILPs degradation in vitro. Recombinant and purified ILPs-HA were co-incubated with IDE or IDE and Aβ peptide for 12 h before western blotting analyses. **b** ILPs inhibit the Aβ degradation in vitro. Aβ peptides were incubated with IDE in the presence or absence of ILPs-HA, and the levels of Aβ were analyzed by western blotting. **c** Aβ and ILPs can be degraded by protein extracts from the control and IDE-overexpression flies. ILP1-HA, ILP5-HA, ILP2-HA and Aβ were incubated with the protein extracts from control and IDE overexpression flies. After incubation, levels of ILPs and Aβ were analyzed by western blotting. Actin was used as the loading control. *Elav-Gal4* was used to drive the overexpression of fly IDE in the CNS. Protein extracts were prepared from fly heads. **d** Aβ inhibits the semi-in-vitro degradation of ILP1 and ILP2. ILP1-HA and ILP2-HA proteins were co-incubated with fly extracts in the presence or absence of Aβ. Levels of ILP1 and ILP2 was analyzed by western blotting. **e** ILPs inhibit the semi-in-vitro degradation of Aβ. Aβ peptides were co-incubated with fly extracts in the presence or absence of ILP1-HA, ILP2-HA and ILP5-HA. Levels of Aβ were analyzed by western blotting. **f** 6bk inhibits the effects of IDE-overexpression on ILP1 and Aβ degradation in vitro. ILP1-HA and Aβ peptides were co-incubated with fly extracts in the presence or absence of 6bk. Levels of Aβ and ILP1-HA were analyzed by western blotting. Uncropped images of the western blots (**a**–**f**) are presented in Supplementary Fig. 12a-Supplementary Fig. 12f. **g** Bovine insulin inhibited the Aβ degradation in vitro. Recombinant and purified IDE protein was used to perform the in vitro cleavage assay. Aβ was co-incubated with IDE or IDE and bovine insulin for 12 h before western analyses. b-insulin bovine insulin. All western blot results here are repeated three times, and only one is shown. *p < 0.05, **p < 0.01, ***p < 0.001

As insulin and Aβ are both substrates of IDE, a hypothesis relating insulin, Aβ and IDE has been proposed, but never tested^17^. According to this model, IDE can degrade Aβ and insulin, and these two substrates may compete for the degrading enzyme. To address this possibility, direct competition between Aβ and *Drosophila* ILPs was tested by an in vitro degradation assay wherein *Escherichia coli*-expressed *Drosophila* IDE and full-length ILPs proteins were co-incubated. These purified ILPs were capable of functionally inducing insulin signaling in vitro to some extent (Supplementary Table 2 and Supplementary Fig. 6). Competition experiments did indeed show that *Drosophila* IDE could degrade both *Drosophila* ILPs (ILP1, ILP2, and ILP5) and Aβ in vitro (Fig. 5a, b). Moreover, Aβ inhibited the degradation of fly ILPs by IDE (Fig. 5a), and conversely fly ILPs (like ILP1, ILP2, and ILP5) inhibited Aβ degradation (Fig. 5b). A subsequent semi-in-vitro degradation assay, wherein protein extracts from *IDE* overexpression flies and control flies were co-incubated with Aβ and fly ILPs, further supported this conclusion. Although both Aβ and fly ILPs could be degraded by WT fly extracts, the extract from *IDE*-overexpressing flies showed enhanced degrading activity (Fig. 5c). Furthermore, Aβ and ILPs antagonized degradation of each other (Fig. 5d, e). The degradation of ILP1 and ILP2 by fly extract was partially blocked by the addition of Aβ (Fig. 5d). Similarly, Aβ degradation by fly protein extract could be largely inhibited by ILPs addition (Fig. 5e). Interestingly, ILPs and Aβ could not completely block the degradation of each other, indicating there are other degrading enzymes in the fly protein extract.

In order to prove that the degradation was due to the function of IDE, 6bk, a compound that inhibits IDE^36^, was used. Degradation of ILP1 and Aβ by the protein extracts was indeed blocked by 6bk (Fig. 5f), especially in the case of the IDE-overexpressed extract. Altogether, these results show that Aβ and *Drosophila* ILPs compete for the activity of *Drosophila* IDE, resulting in reciprocal accumulation. Accumulation of ILPs caused by Aβ could also explain why insulin and insulin signaling were elevated in Aβ flies.

Considering that our recombinant ILPs are not mature in nature and fly in origin, we further tested competition between bovine mature insulin and human Aβ for the action of IDE. The result indicated that bovine insulin could inhibit Aβ degradation by fly IDE protein in vitro (Fig. 5g).

### IDE regulates Aβ toxicity via insulin signaling

**Fig. 6:**
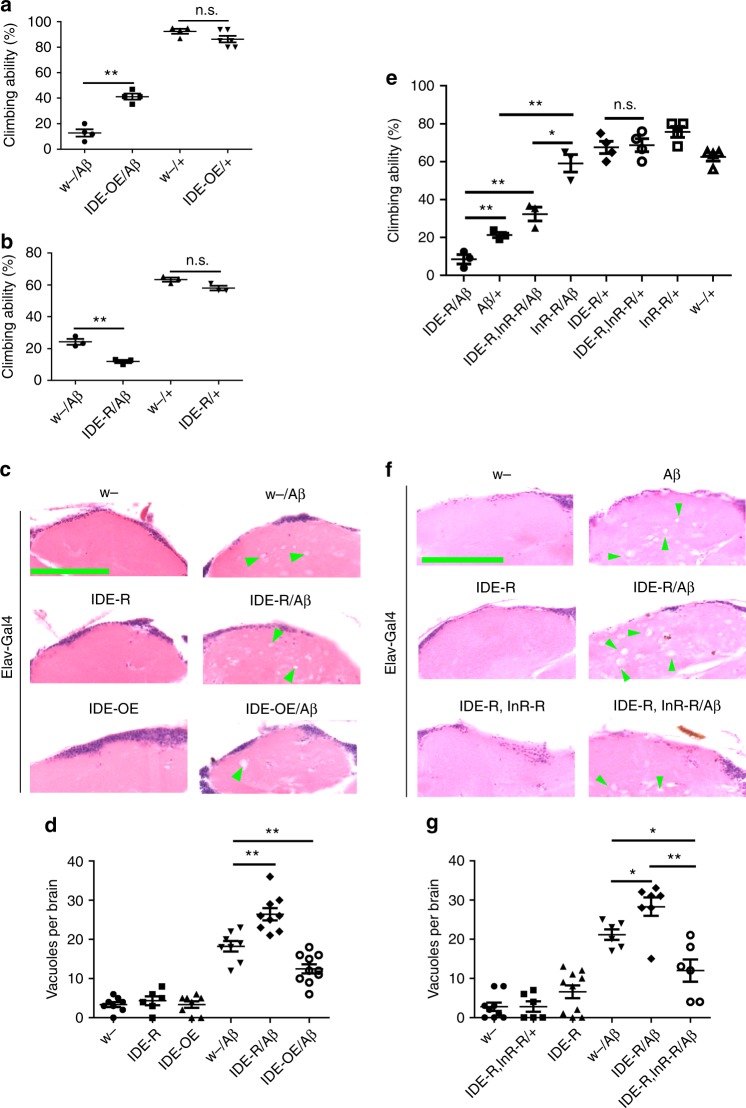
IDE-insulin/InR signaling regulates Aβ toxicity in vivo. **a** Overexpression of *Drosophila IDE* improved the climbing ability of Aβ flies. Data represent mean ± SEM, **p < 0.01. *Elav-Gal4* was used to drive Aβ and fly *IDE* expression in fly CNS. OE overexpression. **b** Knocking-down *Drosophila IDE* exacerbated the climbing disability of Aβ flies. *IDE-R* RNAi of *Drosophila IDE*. *IDE* RNAi was driven by *Elav-Gal4*. Data represent mean ± SEM, **p < 0.01. **c**, **d** Manipulation of the level of IDE affected the degenerative process in the brain of Aβ flies. **c** Hematoxylin and eosin (H&E)-stained paraffin brain sections of these Aβ, Aβ*/IDE-OE*, and Aβ*/IDE-RNAi* flies. *Elav-Gal4* was used to drive gene overexpression or knocking-down in fly CNS. Green arrow heads indicate only some of the many degenerative vacuoles in the brain. Scale bar: 50 μm. **d** The quantification of (**c**). Data represent mean ± SEM, **p < 0.01. **e** The effect of *IDE* and *InR* double RNAi on the climbing ability of Aβ flies. *Elav-Gal4* was used to overexpress and knock-down the corresponding genes. Data represent mean ± SEM, *p < 0.05, **p < 0.01. **f**, **g** H&E-stained paraffin brain sections of the *IDE* and *InR* double RNAi, Aβ-expression flies. Green arrow heads indicate the degenerative vacuoles in flies’ brains. Scale bar: 50 μm. **g** The quantification of (**f**). Data represent mean ± SEM, *p < 0.05, **p < 0.01

As fly IDE could regulate both of the levels of Aβ and ILPs, we wanted to know whether fly IDE could regulate Aβ toxicity in vivo, and whether the effect is through the InR signaling axis. To test whether fly *IDE* can regulate the toxicity of Aβ, *IDE* overexpression and *IDE* RNAi transgenes were introduced into Aβ flies. Overexpression of *Drosophila IDE* significantly rescued the climbing disability of Aβ flies (Fig. 6a); the climbing ability increased from 17% to 42%, whereas no significant difference was found between *IDE*-overexpressing flies and control flies (Fig. 6a). On the other hand, knocking-down *IDE* further reduced the climbing ability of Aβ flies (Fig. 6b); the index was reduced by about ~50%, whereas the effect of *IDE* RNAi by itself on controls was not significant (Fig. 6b), indicating that Aβ toxicity was elevated when fly *IDE* was downregulated.

We then examined whether fly *IDE* had any direct effect on brain degenerative vacuolization due to Aβ expression in the fly CNS. *IDE* overexpression could partially ameliorate the severe vacuolization of Aβ flies (Fig. 6c, d). Consistently, *IDE* knock-down aggravated the vacuolization rate in the brains of Aβ flies (Fig. 6c, d).

To ascertain whether ILPs/InR signaling could mitigate the effects of IDE on Aβ flies, we recombined *IDE* RNAi and *InR* RNAi together in the Aβ fly. *IDE* RNAi reduced the climbing ability of Aβ flies; however, the effect of *IDE* RNAi on Aβ flies was completely suppressed by *InR* RNAi (Fig. 6e). The increase of brain vacuoles caused by *IDE* RNAi was also suppressed by *InR* RNAi (Fig. 6f, g).The epistatic relationship between IDE and InR indicated that the effects of IDE on Aβ toxicity are mediated by the ILPs/InR signaling axis.

### S6K is involved in the action of IDE-ILPs (insulin)/InR on Aβ toxicity

Downstream insulin/InR signaling branches into forkhead box, sub-group O (FOXO) and Ribosomal protein S6 kinase (RpS6K, S6K). It has been shown that activation of insulin signaling may result in FOXO phosphorylation and inactivation^37^. If FOXO inactivation mediates the effects of IDE-insulin/InR on Aβ toxicity, overexpressing FOXO may reduce Aβ toxicity, whereas repressing FOXO may aggravate Aβ toxicity. Therefore, we tested whether overexpression and RNAi of fly FOXO could change the toxicity of Aβ. Unfortunately, overexpression of FOXO in the fly CNS led to larval death at the first instar stage. Similarly, overexpression of FOXO-GFP (green fluorescence protein) in the fly CNS also caused developmental arrest, with many flies dying on the pupal stage. Nevertheless, some of the FOXO-GFP-expressing flies did manage to eclose, although with abnormal wing posture. Importantly, expression of FOXO-GFP in the fly CNS did not alter the climbing abnormality of these eclosed Aβ flies despite their abnormal wing posture (Supplementary Fig. 7a). Knock-down of FOXO or expression of the phosphorylated form of FOXO did not aggravate the toxicity of Aβ (Supplementary Fig. 7b); instead, they both reduced the climbing abnormalities of Aβ flies. Altogether, these results suggest that FOXO inactivation may not boost Aβ toxicity. If anything, FOXO inactivation mitigates Aβ toxicity, likely in an indirect manner.

**Fig. 7:**
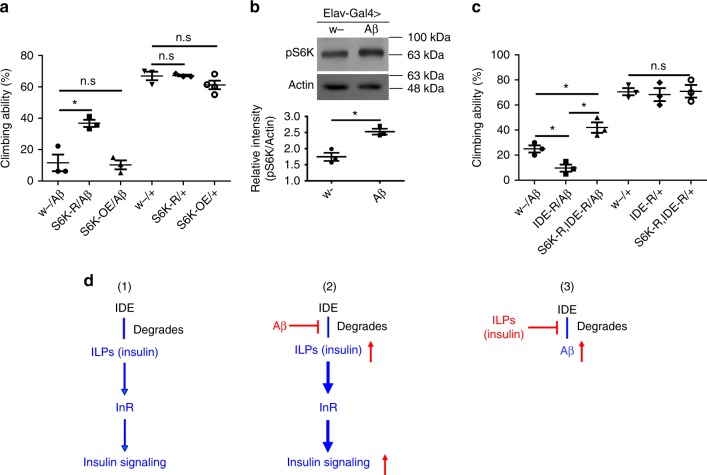
IDE-insulin/InR-S6K signaling mediates Aβ toxicity. **a** Effects of *S6K* manipulation on the climbing ability of Aβ flies. Data represent mean ± SEM, *p < 0.05. *Elav-Gal4* was used to drive Aβ and genes OE or RNAi in fly CNS. OE overexpression. **b** The levels of phosphorylated S6K in Aβ flies were analyzed by western blotting (uncropped images of the blots are presented in Supplementary Fig. 12g). Actin was used as the loading control. Results are reproducible in three independent western blotting experiments, and only one is shown here. *p < 0.05. **c** S6K RNAi reduced the effect of IDE RNAi on the climbing ability of Aβ flies. Data represent mean ± SEM, *p < 0.05. **d** The relationship between Aβ toxicity and insulin pathway. (1–3) Local insulin pathway is a major mediator of Aβ toxicity. IDE can degrade both insulin and Aβ peptides. Insulin and Aβ therefore competes for the enzymatic activity of IDE, resulting in reciprocal inhibition. (1) Normal condition: IDE degrades ILPs (insulin). (2) Aβ inhibits the degradation of insulin. In the Aβ flies, accumulation of Aβ will increase the level of insulin and consequently causes activation of downstream insulin signaling, including S6K activation. Therefore, downregulation of insulin and insulin signaling can effectively reduce the toxicity of Aβ. (3) Insulin inhibits the degradation of Aβ, leading to increased level of Aβ

S6K is another downstream branched effector of insulin signaling, also involved in the aging process^38^. S6K is activated when insulin signaling activity is elevated. We tested whether S6K was involved in IDE-ILPs (insulin)/InR’s effects on Aβ toxicity. Our results indicated *S6K* knock-down improved the climbing ability of Aβ flies (Fig. 7a). Brain sections also revealed S6K manipulation could reduce Aβ toxicity in the flies (Fig. 2c, d). Furthermore, western blot revealed that the level of phosphorylated S6K (p-S6K) was elevated in Aβ flies (Fig. 7b), indicating S6K was activated in Aβ flies, consistent with the elevation of fly insulin signaling.

To ascertain whether S6K could mitigate the effect of IDE on Aβ flies, we introduced *IDE* RNAi and *S6K* RNAi into the Aβ fly. *IDE* RNAi reduced the climbing ability of Aβ flies. However, the effect of *IDE* RNAi on Aβ was suppressed by *S6K* RNAi (Fig. 7c): S6K RNAi improved the climbing ability upon IDE knock-down, indicating that S6K mediates the effect of IDE on Aβ toxicity and this IDE-ILPs (insulin)/InR-S6K signaling axis is involved in Aβ toxicity. Consistently*, chico* RNAi decreased the level of p-S6K in Aβ flies (Supplementary Fig. 8). Interestingly, when *mth* or *Indy* were knocked-down, the level of p-S6K was also reduced in Aβ flies (Supplementary Fig. 8), indicating S6K may also be involved in the effects of *mth* and *Indy* on Aβ toxicity, and in the crosstalk between *mth* and insulin signaling.

## Discussion

Mammalian insulin/insulin signaling is a complex process. The role and action of mammalian insulin/Insulin-like growth factor 1 (IGF-1) in the brain is not fully understood. Changes in insulin signaling may not only affect InR signaling, but also IGF-1 receptor signaling^39^. The model organism *Drosophila* is endowed with a comparably simpler insulin/insulin signaling pathway, which may facilitate understanding of the connection between AD and insulin/insulin signaling; in particular, the connection between toxicity of Aβ and insulin/insulin signaling.

In this work, we presented evidence that Aβ and ILPs compete for fly IDE activity and that Aβ causes accumulation of fly ILPs and elevation of fly InR-S6K signaling. This IDE-ILPs (insulin)/InR-S6K signaling axis is involved in Aβ toxicity, and knocking-down of ILPs, InR or the downstream components S6K is an effective means to suppress Aβ toxicity (Fig. 7d, model). As insulin signaling is an ageing factor, our results help to connect several important phenotypes, namely ageing, AD and diabetes, through insulin signaling.

The role of insulin and insulin signaling in human AD is debated. It is not fully clear whether the alteration of insulin signaling is the cause or the consequence of AD^26^. Decreased insulin signaling occurs in patients with type II diabetes, where higher insulin levels are often elicited, likely as a compensatory action. These patients overall have a higher incidence of AD. On the other hand, it has been reported that insulin levels in AD patients are higher than in healthy persons^15^. Interestingly, clinical insulin treatment is associated with increased AD rate^10,16–18^. How insulin and insulin signaling connect with AD mechanistically is confusing. Is decreased insulin signaling sensitivity such as in diabetes a cause of AD increase? Or is elevated insulin signaling related to AD? Our results strongly indicate that reduction of insulin and insulin signaling can effectively reduce, instead of aggravate, Aβ pathology (Fig. 7d, model), favoring the latter possibility.

It has been reported that AD brains tend to be insulin resistant, accompanied by decline of insulin signaling. The decline of insulin signaling has been proposed as a consequence of AD and a cause further aggravating AD pathology^26^. However, our observation that reducing insulin signaling can reduce, instead of aggravate, toxicity of Aβ suggests an alternative explanation: the association of insulin resistance or decrease of insulin signaling with AD may be considered a protective response to minimize the damage from Aβ. Considering that reduction of insulin signaling can improve cell survival and activation can trigger cell death, reduction of insulin signaling as a protective approach to avoid the more severe damage and prolong cell survival in AD brain seems reasonable.

It has been reported that IDE activity is reduced in some tissues during the aging process^30,40^ and it is connected with AD pathology. Reducing the level of IDE or genetic variations of *IDE* alter AD pathology^30,41,42^. In mouse models, Aβ level was elevated when IDE was knocked out, and AD-related pathology was exacerbated under that condition^32–34^. In our hands, we found that while manipulation of IDE could reduce Aβ toxicity, knock-down of the insulin receptor and some of its downstream components could to a much greater extent suppress the defects of the Aβ flies and the effects of IDE RNAi. In the absence of Aβ, knocking-down *IDE* by itself could cause some deleterious effects, such as the drop of climbing ability in aged flies (Supplementary Fig. 9), consistent with the notion that accumulation of ILPs/insulin and other substrates is a central theme not only to the pathophysiology of Aβ flies, but also to some aspects of ageing in normal flies. Besides IDE, other factors like Neprilysin (NEP) might also contribute to the degradation of insulin and Aβ^43^. This explains why sole manipulation of IDE level does not affect Aβ toxicity as significantly as that of *Drosophila* ILPs (insulin), InR and some of other downstream signaling components.

**Fig. 8:**
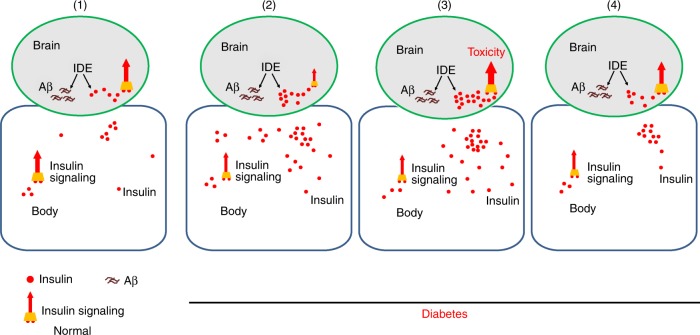
A hypothetical model to explain the potential connection between diabetes and AD. We propose that in some diabetes patients with AD, overall reduced insulin sensitivity may not necessarily apply to the pathological areas of their brains. Instead, systemic compensation or drug intervention in the body might enhance regional brain insulin signaling and aggravate AD progression. (1) Brain insulin signaling under normal condition. At this state, the brain local insulin and systemic insulin signaling are in balance, i.e., both are at normal rate. (2–4) Brain insulin signaling under diabetes condition and its influences on Aβ toxicity. The diabetes patients can be theoretically divided into three categories. (2) In this category of diabetes patients, brain insulin signaling may be more affected than the body (more decreased in the local brain). Raising insulin signaling to a level that can compensate for the body loss may still not be enough to compensate completely for the signal loss in the brain. These patients may have protection from the toxicity of Aβ because their brain insulin signaling is less than normal even after the increase. (3) In this category, brain local insulin signaling may not be as affected as the rest of the body. Increasing systemic insulin to a level that can compensate the body loss may overcompensate the signaling loss in the brain. These patients will be more vulnerable to AD. (4) In this category, brain insulin signaling components are similarly affected as the body. The increased insulin just roughly compensates the loss in the affected brain resulting similar insulin signaling as the normal. In this group of diabetes patients, AD rate will not be affected

It appears therefore that the involvement of insulin signaling in human AD pathology may consist of a complex repertoire of different states (Fig. 8, model), which may explain the conflicting results in this field. In some or probably most AD patients, insulin signaling is not affected. In some AD patients, insulin signaling is reduced overall accompanied by type 2 diabetes. However, we speculate that in some regions of the brain insulin signaling is not affected as much as in the rest of the body. In this scenario, compensatory insulin secretion in the rest of the body or insulin treatment will more than offset the loss of insulin signaling in the brain and exacerbate Aβ toxicity. In still some other AD patients, insulin signaling in the brain may be comparably more severely affected than in the rest of the body, affording a protective measure. In other words, compensation of the signaling loss in the body will not be enough to remedy the brain signaling loss in this case.

According to our RNAi-based genetic screening results, besides the insulin signaling component *chico*, the other two aging-related genes, *mth* and *Indy*, could also reduce the toxicity of Aβ. The mechanism of lifespan extension by *mth* and *Indy* remains to be elucidated. However, some evidence suggests that *mth* and *Indy* may crosstalk with insulin signaling^44,45^: Mth and its ligand Sun (stunted) can modulate physiological insulin levels in response to nutrient inputs in the larvae, helping to understand the crosstalk between mth and insulin signaling; decreasing Indy levels may be coupled with reduced insulin signaling. From this perspective, it is possible that Mth and Indy act through the insulin connection to modulate Aβ toxicity, and indeed we found p-S6K was decreased in *mth* and *Indy* RNAi flies. Nevertheless, how big an effect this is, i.e., whether Mth and Indy regulate Aβ toxicity entirely or mainly through insulin signaling, is unclear.

It has been suggested that downstream insulin signaling in AD may switch to mammalian target of rapamycin (mTOR) signaling^46^. Our results indicate that S6K is at least partially involved in Aβ pathology, and mediates the effects of IDE-insulin/InR signaling on Aβ toxicity. The downstream targets of insulin and insulin signaling remain incompletely elucidated^47,48^. At present, we still do not know how many downstream components of insulin signaling are involved in AD. Some results are still controversial and unclarified due to the complexity of insulin actions in mammals^17,49^. Our results indicate that FOXO inactivation may not be involved in insulin action on Aβ toxicity. Interestingly and paradoxically, it has been reported that, instead of inactivation, FOXO is activated by Aβ and mediates Aβ’s effect on cell death through Bim^50^, and this activation is likely caused by oxidative stress^51,52^, which is elevated by Aβ^53^. Our results indicate S6K, activated in AD flies, is involved and contributes to the pathological process of Aβ. Supporting this, it was also reported that S6K is activated in AD patients^54^. The mechanism of lifespan extension by S6K inhibition is thought to involve AMPK activation^38,55^, consistent with our results showing that AMPK inhibition can enhance Aβ toxicity (climbing disability, Supplementary Fig. 10).

In summary, we demonstrated that brain IDE-ILPs (insulin)/InR-S6K signaling is involved in AD pathology, which implies the mechanism contributing to ageing-related AD may be the same as the cause of ageing in the brain. These experimental findings offer better understanding of AD, ageing and diabetes, and may provide new AD-relevant strategies and therapeutic targets in the future.

## Methods

### Fly stocks and genetics

Fly stocks used in this study were mainly obtained from the Bloomington *Drosophila* Stock Center, the Vienna *Drosophila* Resource Center (VDRC), and the *Drosophila* Stock Center of Tsinghua University. The RNAi flies of *chico* (*CG5686*), *Tor* (*CG5092*), and *Pi3K68D* (*CG11621*), *Pi3K21B* (*CG2699*), *ILP1* (*CG14173*), *ILP2* (*CG8167*), *ILP3* (*CG14167*), and *ILP5* (*CG33273*) were obtained from Tsinghua *Drosophila* Stock Center. *UAS-InR* fly and RNAi flies of *InR* (*CG18402*) (line 1# and line 2#), *UAS-FOXO (CG3143)*, *UAS-FOXO* RNAi, *UAS-pFOXO*, *UAS-FOXO-GFP. Flag*, *UAS-S6K* (*CG10539*), and *S6K* RNAi flies were obtained from the Bloomington *Drosophila* Stock Center. *IDE* (*CG5517*) RNAi fly was obtained from the VDRC. IDE.GFP (expression GFP under the control of the fly IDE promoter) and *UAS-IDE* fly and the corresponding background *w*^−^ flies^35^ was a kind gift from Dr. Pablo Wappnera (Universidad de Buenos Aires). *Elav-geneswitch* and *UAS-Aβ42* fly was from Dr. Yi. Zhong (Tsinghua University). Control WT flies corresponded, respectively, to the background flies of each resource, and were labeled as WT or w^−^.

In order to introduce various transgenes to Aβ fly background, *UAS-Aβ* flies were first crossed with *Elav-Gal4* flies to obtain *Elav-Gal4; UAS-Aβ/Cyo*, which were then used to crossed in different transgenes. As the controls, the corresponding background flies of these transgenes were crossed with *Elav-Gal4* or crossed with *Elav-Gal4; UAS-Aβ/Cyo*, to obtain *Elav-Gal4/+* in background flies, or *Elav-Gal4/+; UAS-Aβ/+* in background flies. For the transgenes from VDRC, the VDRC *w*^−^ fly was used as the background fly; for the transgenes from Bloomington *Drosophila* Stock Center, Bloomington *w*^−^ was used as the background; for the transgenes from Tsinghua *Drosophila* Stock Center, the same background fly was used, and for *UAS-IDE*, the corresponding background *w*^−^ fly was used.

Flies were raised with standard corn meal under 25 ( ± 1) °C unless noted. GSK19045297 and Wortmannin were added in standard corn meal food at 5 μM/L final concentration, and the same volume of dimethylsulfoxide was added in the control. RU486-induced expression of Elav-geneswitch was performed as described^56^. RU486 (mifepristone) was used at 20 μg/mL final concentration, and in the control, the same volume of solvent EtOH was added.

### Climbing ability assay

Flies were maintained at 25 °C on standard corn media. The climbing ability assay was as previously described. Flies were aged for ~4 weeks at 25 °C. The climbing ability represented the total number of flies that were able to climb 5 cm in 8 s, divided by the total number of flies in every assayed group (%). For each genotype, at least three individual repeats were assayed. For GSK19045297 and Wortmannin fed flies, flies were aged for ~4 weeks at 25 °C, and then assayed for climbing ability. At least three individual repeats were performed for each drug treated group. For RU486 fed flies, their climbing ability was defined as the total number of flies able to climb 7 cm in 8 s, divided by the total number of flies in every assayed group (%).

### Fly protein extraction and western blot

For AKT and ILP5 hybridization, at least 30 adult heads (aged for 4 weeks) were collected and homogenized in NP40 lysis buffer containing protease inhibitors and phenylmethane sulfonyl fluoride. After centrifugation at 12,000 *g*, 4 °C for 10 min, the supernatants were collected, mixed with sodium dodecyl sulfate (SDS) loading buffer and then heated at 75 °C. After centrifugation at 10,000 *g* for 5 min, the protein samples were loaded into 15% SDS–polyacrylamide gel electrophoresis (SDS-PAGE) gel for electrophoresis separation. Separated proteins were transferred onto polyvinylidene fluoride (PVDF) membranes (Millipore) and hybridized with antibodies after blocking with 5% milk in phosphate buffered saline with Tween 20 (PBST) buffer.

AKT and p-AKT antibodies were generously provided by Xun Huang’s Lab (Institute of Genetics and Developmental Biology, Chinese Academy of Sciences, Beijing, China). Actin antibody was purchased from ZSGB-BIO company (China), Tubulin and ILP5 antibodies were purchased from Abcam company (UK), ILP2 antibody was purchased from Abmart (Shanghai, China), and p-S6K antibody was purchased from Cell Signaling Technology company (CST, USA). Secondary antibodies were peroxidase-labeled anti-mouse IgG, or anti-rabbit IgG (CST, USA). Immunoblot signals were developed by enhanced chemiluminescence (Pierce, USA).

Soluble and insoluble Aβ fractions were separated as described^57^. Briefly, 50 fly heads were collected and homogenized in NP40 lysis buffer (containing 1% SDS), centrifuged at 10000 *g* to remove the debris. The supernatants were collected and centrifuged again at 10,0000 *g* for 30 min. The supernatants (soluble fractions) were then mixed with SDS loading buffer, and the remaining pellets (the insoluble fractions) were solubilized by 70% formic acid and later sonicated to break up the Aβ aggregates. After evaporating the formic acid, the protein samples were dissolved in 2 × SDS loading buffer. Both the soluble and insoluble fractions were heated at 75 °C before loading onto the PVDF membrane and hybridizing with the anti-Aβ42 antibody (Cell Signaling Technology, USA), or running into the 15% SDS-PAGE gel for western blot. Similar procedures were followed with AKT and ILP5 western blot. The intensity of gel bands was quantitated using ImageJ (U.S. National Institutes of Health, Bethesda, MD, USA).

### *Drosophila* head sectioning and H&E staining

*Drosophila* head sectioning and H&E staining were performed as previously described^58^. Briefly, before the experiment, fly were aged for 4 weeks, fly heads were collected and fixed with Carnoy fixation solution (ethanol: chloroform: acetic acid = 6:3:1) for 4 h, and the samples were then dehydrated twice by 100% ethanol for 30 min, once by dry ethanol (100% ethanol dried with desiccant) for 1 h, and once by methyl benzoate for 1 h, before embedding into melted paraffin. The embedded fly heads were sectioned into 8 μm continuous sections using the Leica section apparatus (RM2235, Germany). H&E (ZSGB-BIO, China) staining was used to facilitate the observation of the vacuoles in the brains.

### Constructs, protein expression, purification, and in vitro degradation assay

*Drosophila ILP1*, *ILP2*, *ILP5*, and *IDE* gene coding regions were amplified from fly complementary DNA (cDNA), and cloned into the pMXB10 plasmid (NEB, New England Biolabs). Protein expression and purification were performed as previously described^58^. Briefly, proteins were purified with chitin beads (NEB) as described by vendor brochure, and finally dissolved in 0.1 M PBS (pH = 7.4) buffer containing 1 mM dithiothreitol (DTT) after desalt treatment using the Zeba™ desalt column (ThermoFisher Scientific). In vitro degradation assay was performed by adding the purified proteins and the Aβ peptide (from Yanmei Li’s Lab, Tsinghua University, 0.1 M PBS, pH = 7.4), mixing and overnight incubation. The protein levels were analyzed by western blot. For bovine insulin competitive assay, bovine insulin was purchased from Macklin Company (China).

### Immunostaining

Immunostaining assay was performed as described^59^. Briefly, fly heads were collected and dissected to obtain the brains in phosphate buffered saline (PBS). After fixing in 4% paraformaldehyde, the brains were incubated in PBS containing 0.3% Triton X100 for 30 min, washed 3 times in PBS, and then blocked with 10% normal goat serum for 1 h, before incubating with the primary antibodies overnight (for Aβ immunostaining, the Aβ antibody was diluted with 1:300). Samples were washed with PBST (0.1% Tween 20) for three times, and then incubated with fluorescein isothiocyanate or tetramethylrhodamine-labeled anti-mouse or anti-rabbit IgG for 1.5 h. After washing with PBST (0.1% Tween 20) for three times, fly brains were examined under the confocal microscope (Zeiss 710 META) for image capturing.

### Fly mRNA extraction and RT-PCR

For total head RNA extraction, at least 20 fly heads were collected. To extract total RNA from fly fat body, at least 20 fly abdomens were collected and the fat bodies were separated. Fly mRNA extraction and RT-PCR were performed as previously described^60^. Briefly, total RNA was extracted with TRIzol reagent (Invitrogen, Carlsbad, CA, USA). cDNA was reverse transcribed by the TransScript Reverse Transcriptase (TransGen Biotech Co., Beijing, China). Semiquantitative RT-PCR was performed using gene-specific primers to amplify partial regions of the target genes. RNA isolation, reverse transcription^61^, and semiquantitative RT-PCR were performed independently for three times. The intensity of gel bands was quantitated using ImageJ (U.S. National Institutes of Health, Bethesda, MD, USA). Primer sequences used in RT-PCR are listed in Supplementary Table 3.

### Statistics

Data are presented as mean ± S.E.M. Differences among groups were analyzed by the GraphPad Prism 5 software with Student’s *t*-test (comparison of two groups) or ANOVA test (three groups or more). *p < 0.05, **p < 0.01, ***p < 0.001.

## Supplementary information

Supplementary Information

Supplementary Data 1

Description of Supplementary Data

## Data Availability

The datasets generated and/or analyzed during the current study are available from the authors upon request. The source data underlying the graphs and charts presented in the main figures are presented in Supplementary Data 1.
