# Supplementary material for: Insulin signaling in Drosophila melanogaster mediates Aβ toxicity
Source: Commun Biol. 2019 Jan 8;2:13. doi: 10.1038/s42003-018-0253-x (PMC6325060; doi:10.1038/s42003-018-0253-x)
Supplement: Supplementary file 1 — Supplementary Information [file 42003_2018_253_MOESM1_ESM.docx]

**Supplementary Figure 1**

**
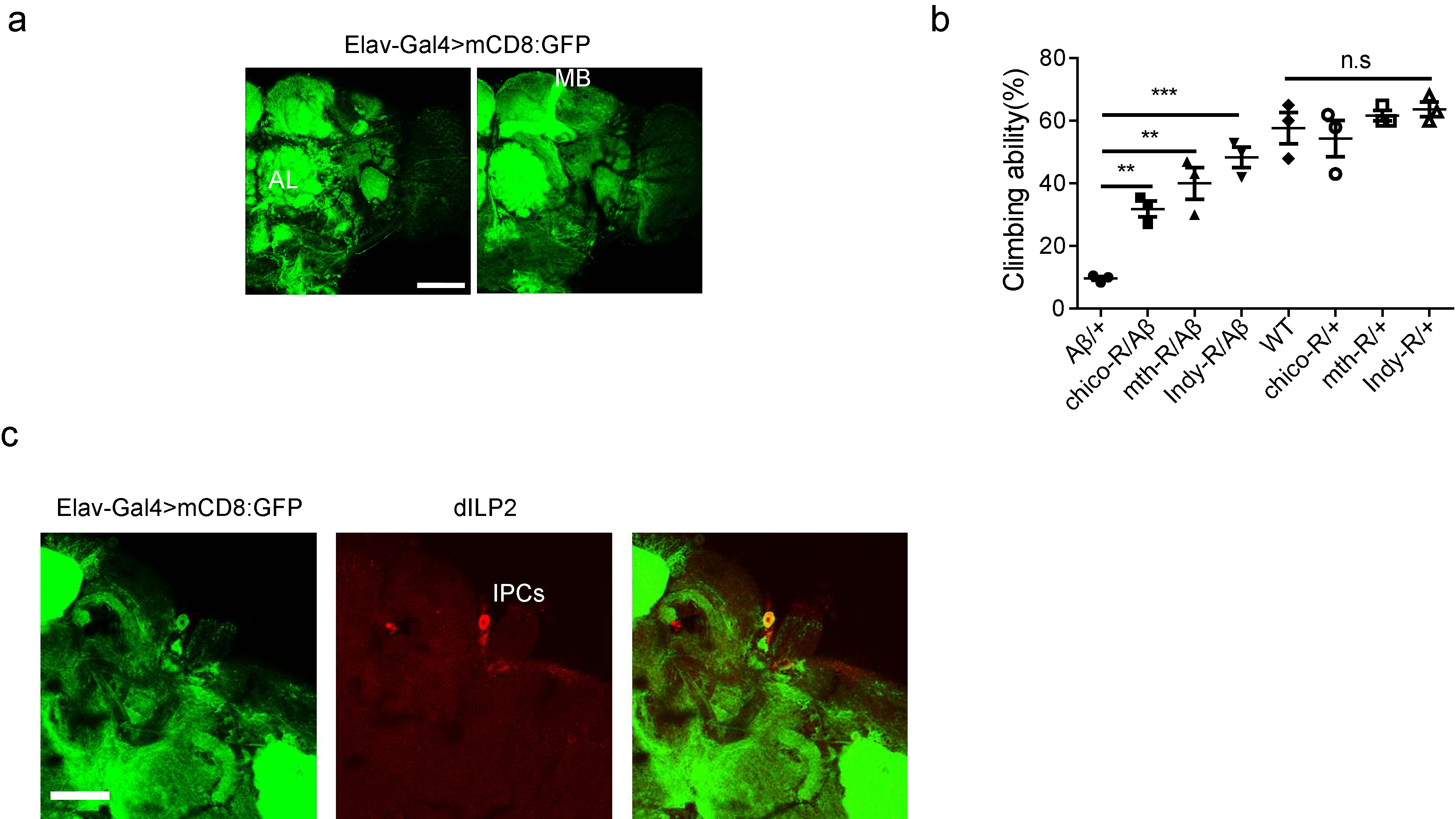
**

**Supplementary Figure 1. Effects of *chico*, *mth*, *Indy* RNAi on the climbing ability of Aβ flies**

(**a**) Elav-Gal4 expresses widely in the adult fly brain. mCD8: GFP was driven by Elav-Gal4. Scale bar=100μm. MB: mushroom body, AL: antennal lobe.

(**b**) Down-regulation of fly *chico*, *mth* and *Indy* improves the climbing ability of Aβ flies. *Elav-Gal4* was used to express Aβ or knockdown of *chico*, *mth* and Indy in the fly CNS; flies were raised at 29 ℃. Data represent mean±SEM, **p<0.01.

(**c**) Elav-Gal4 expresses in insulin producing cells (IPCs). Expression pattern of Elav-Gal4 was marked by mCD8: GFP; IPCs were marked by ILP2 immunostaining. Scale bar=50μm.

**Supplementary Figure 2**

**
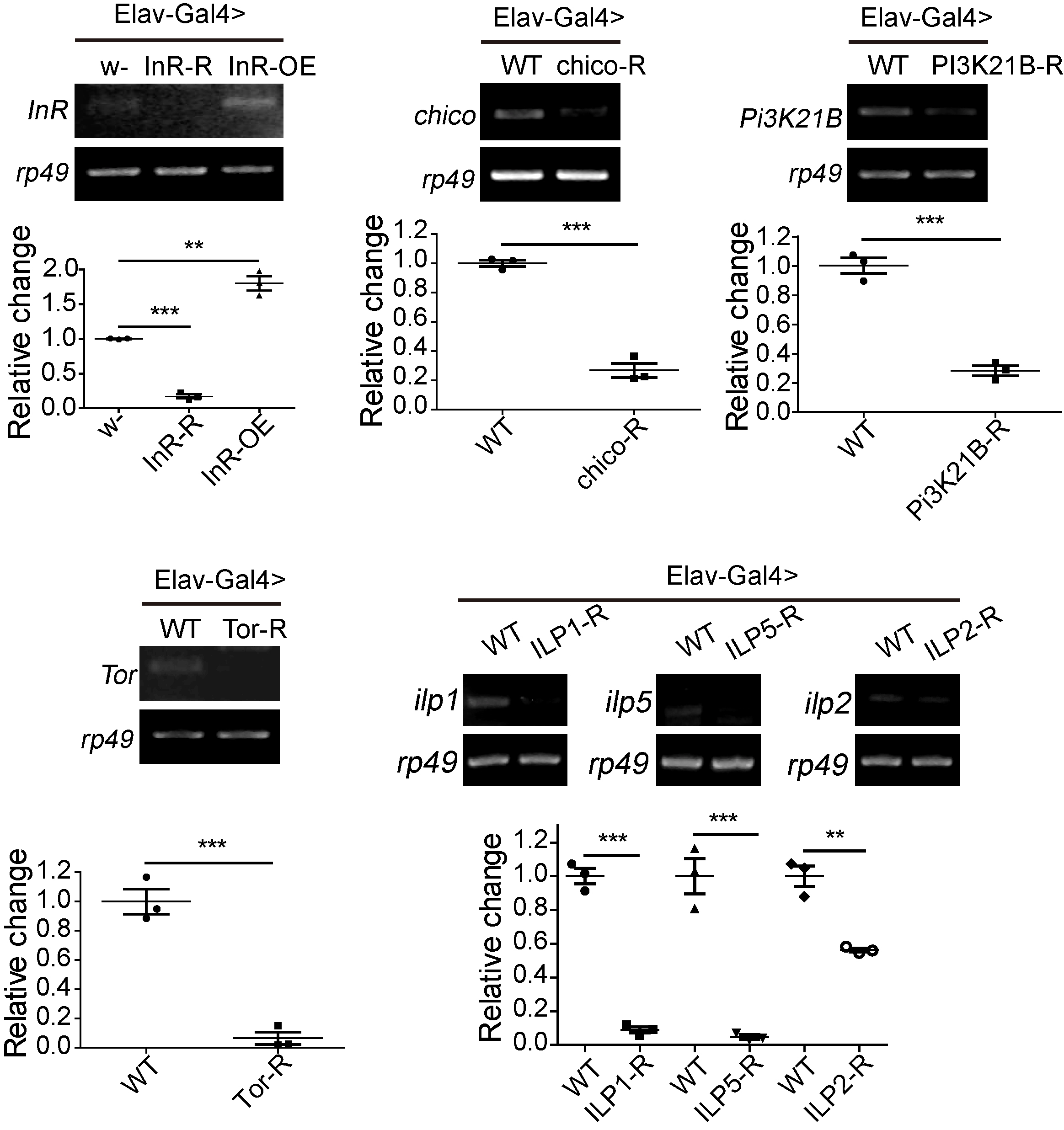
**

**Supplementary Figure 2. RNAi efficiencies of the downstream components of insulin signaling**

RNAi efficiencies were analyzed by RT-PCR. *Elav-Gal4* was used to drive

the overexpression or RNAi of the components of insulin pathway. *rp49* was used as the loading control. Results are reproducible in three independent RT-PCR experiments, and only one is shown here. Data represent mean±SEM, *: p<0.05, **: p<0.01, ***: p<0.001.

**Supplementary Figure 3**

**
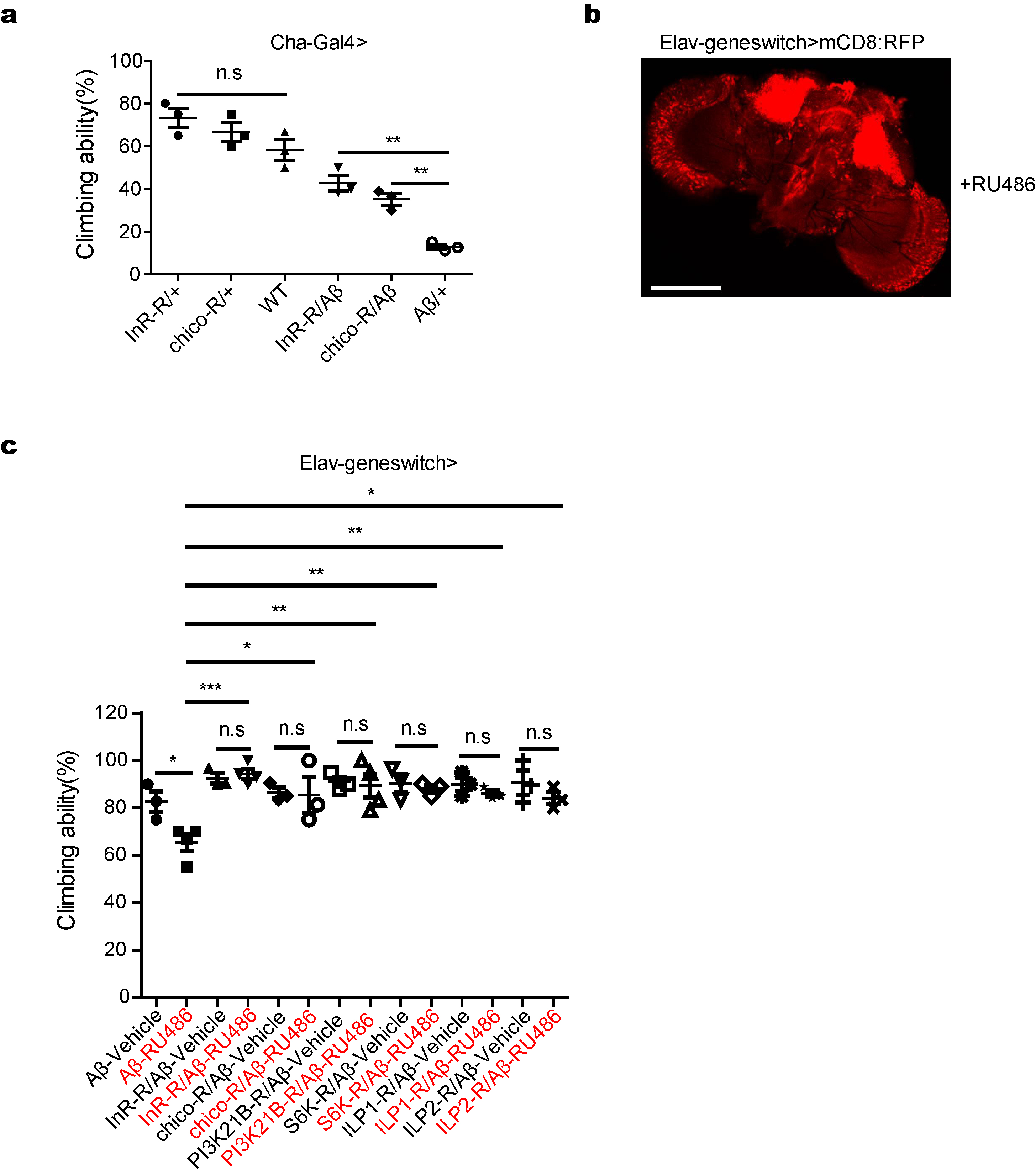
**

**Supplementary Figure 3. Knocking-down the components of insulin signaling by Cha-Gal4 or Elav-geneswitch mediated RNAi suppresses Aβ toxicity.**

**(a)** The climbing ability after *InR* or *chico* was knocked-down in fly cholinergic neurons by Cha-Gal4. **: p<0.005.

**(b)** RU486 activates Elav-geneswitch expression in adult CNS. UAS-mCD8:RFP was used to mark the expression of Elav-geneswitch. Scale bar=200μm.

**(c)** Knocking-down the components of insulin signaling at the adult stage could still suppress Aβ toxicity. Expression of Aβ and knocking-down of the components of insulin signaling were induced by RU486 at the adult stage. Comparison between the climbing abilities of vehicle and RU486 groups, and comparison of the climbing ability of transgenic flies across genotypes were shown here. Data represent mean±SEM , *: p<0.05, **: p<0.005.

**Supplementary Figure 4.**

**
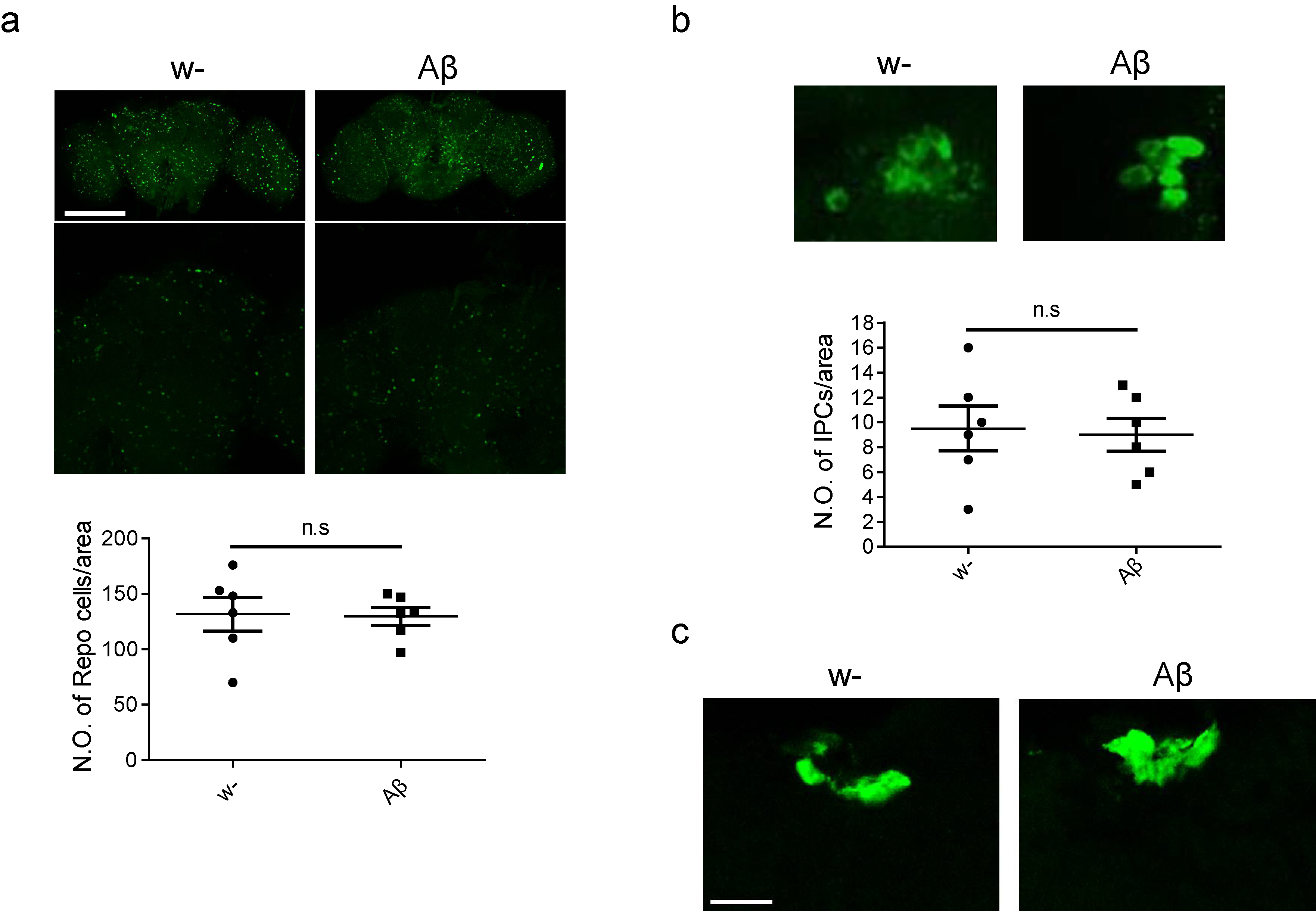
**

**Supplementary Figure 4. Gila cells and IPCs are not affected in AD flies.**

(**a**) Repo staining was performed to mark the repo glia cell. The number of repo-expressing glia cells was counted, scale bar=200μm, and data represent mean+SEM, n>6. *: p<0.05.

(**b**) ILP2 staining of AD fly brains. The number of IPCs was counted, and data represent mean+SEM, n>6. *: p<0.05.

(**c**) ILP2 was accumulated in IPCs as revealed by ILP2 staining. Scale bar=20μm.

**Supplementary Figure 5.**

**
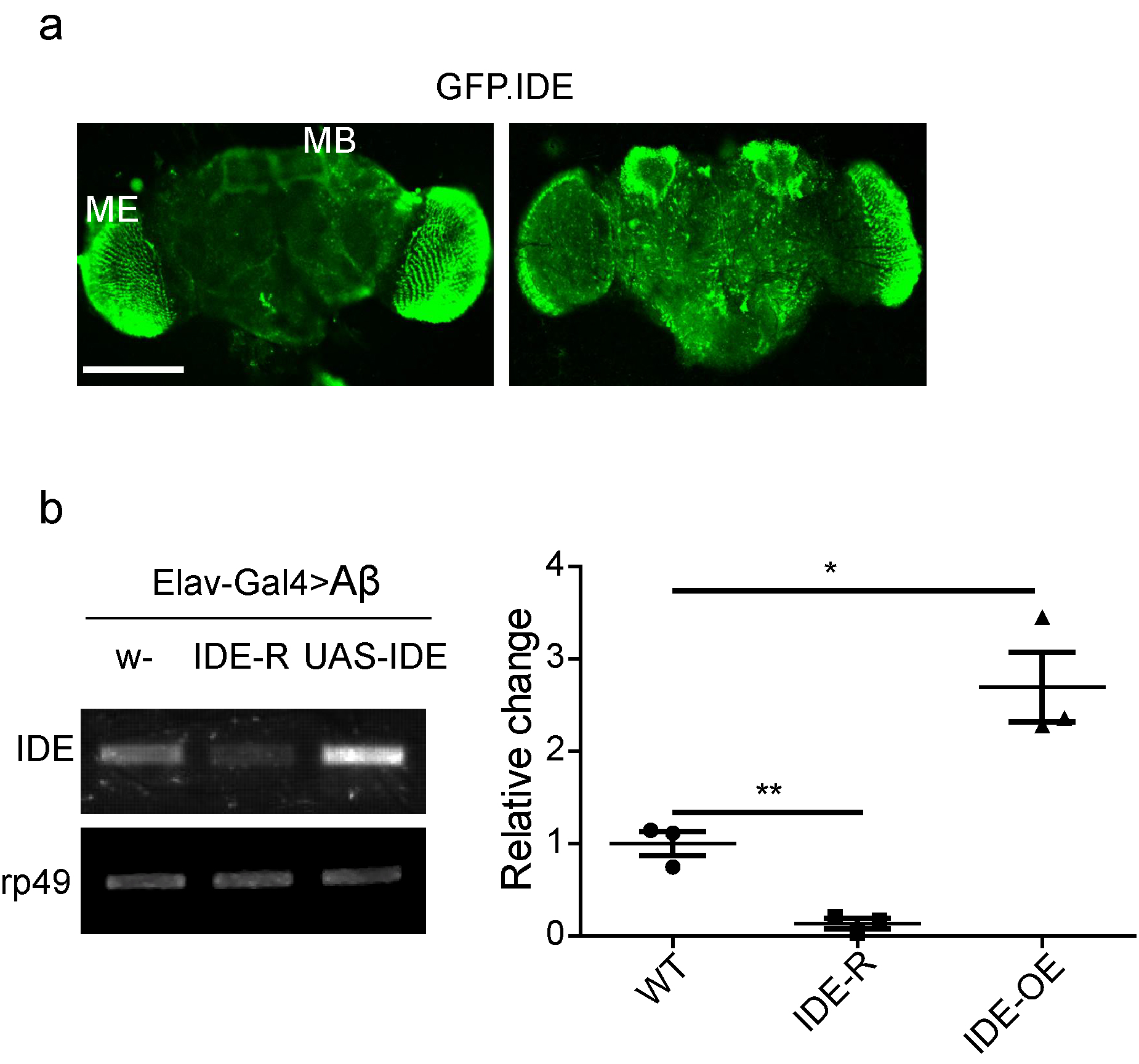
**

**Supplementary Figure 5. The expression pattern of IDE in the adult brain, and the efficiencies of *IDE* overexpression and RNAi**

**(a)** IDE is widely expressed in the adult fly brain, including CNS. IDE.GFP (expression GFP under the control of the fly IDE promoter) was used to indicate the expression pattern of fly IDE in the adult brain. Scale bar=200μm. MB: mushroom body, ME: medulla.

**(b)** The efficiencies of *IDE* overexpression and RNAi. mRNA levels were analyzed by RT-PCR. *Elav-Gal4* was used to drive *IDE* overexpression or knockdown in the fly CNS. Results are reproducible in three independent RT-PCR experiments, and only one is shown here. Data represent mean±SEM , *: p<0.05, **: p<0.01.

**Supplementary Figure 6**

**
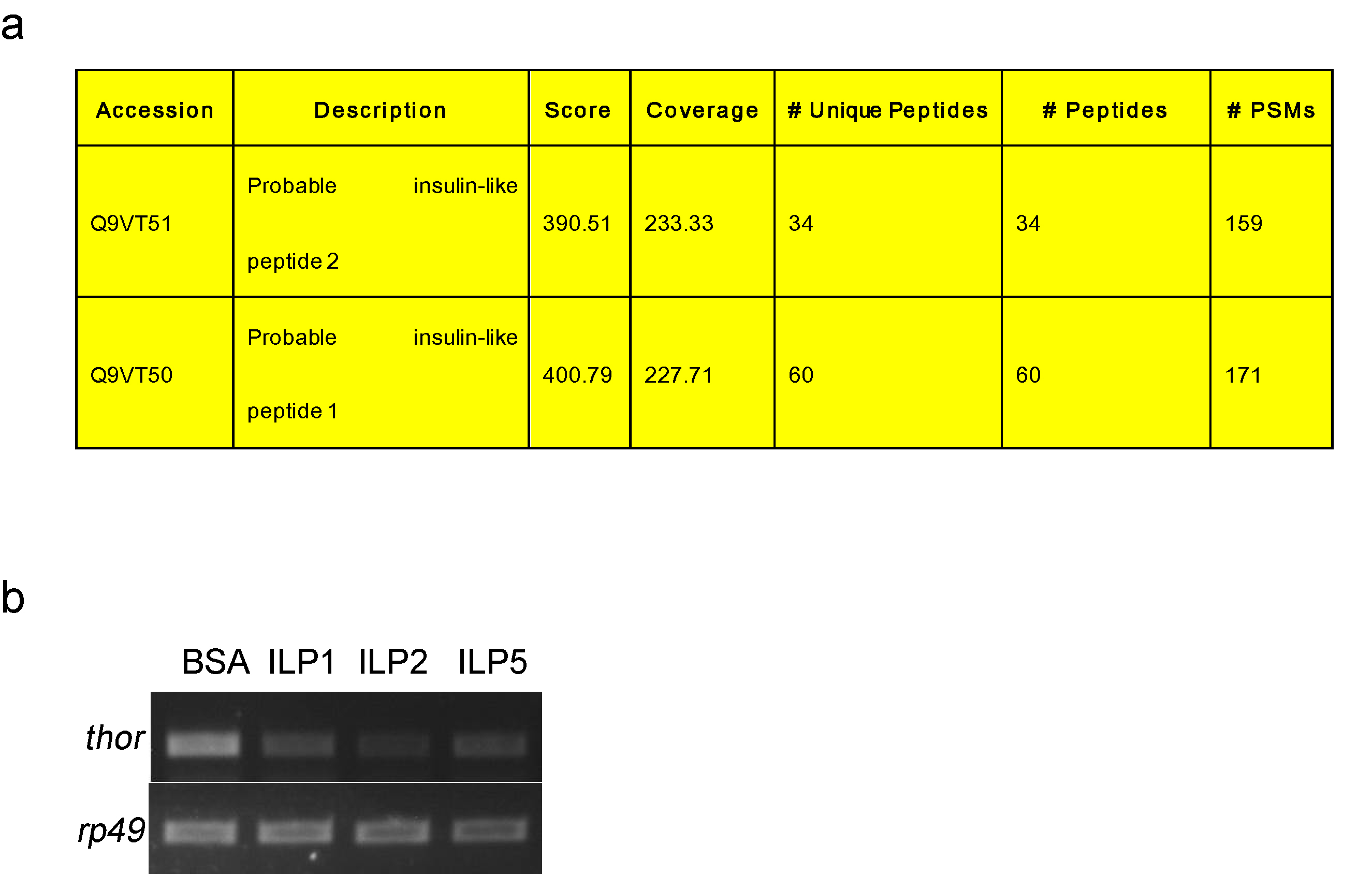
**

**Supplementary Figure 6. Purified ILP1, 2 and 5 proteins are functional.**

ILP1-HA, ILP2-HA and ILP5-HA could induce insulin signaling in the fat body *in vitro*. Fat bodies form WT 3^rd^ instar larvae were cultured in S2 medium, and ILP1, 2 and 5 proteins were added in respectively. After overnight incubation, *thor* mRNA level was examined to indicate the activity of insulin signaling in the fat body. Rp49 was used as the loading control.

**Supplementary Figure 7.**

**
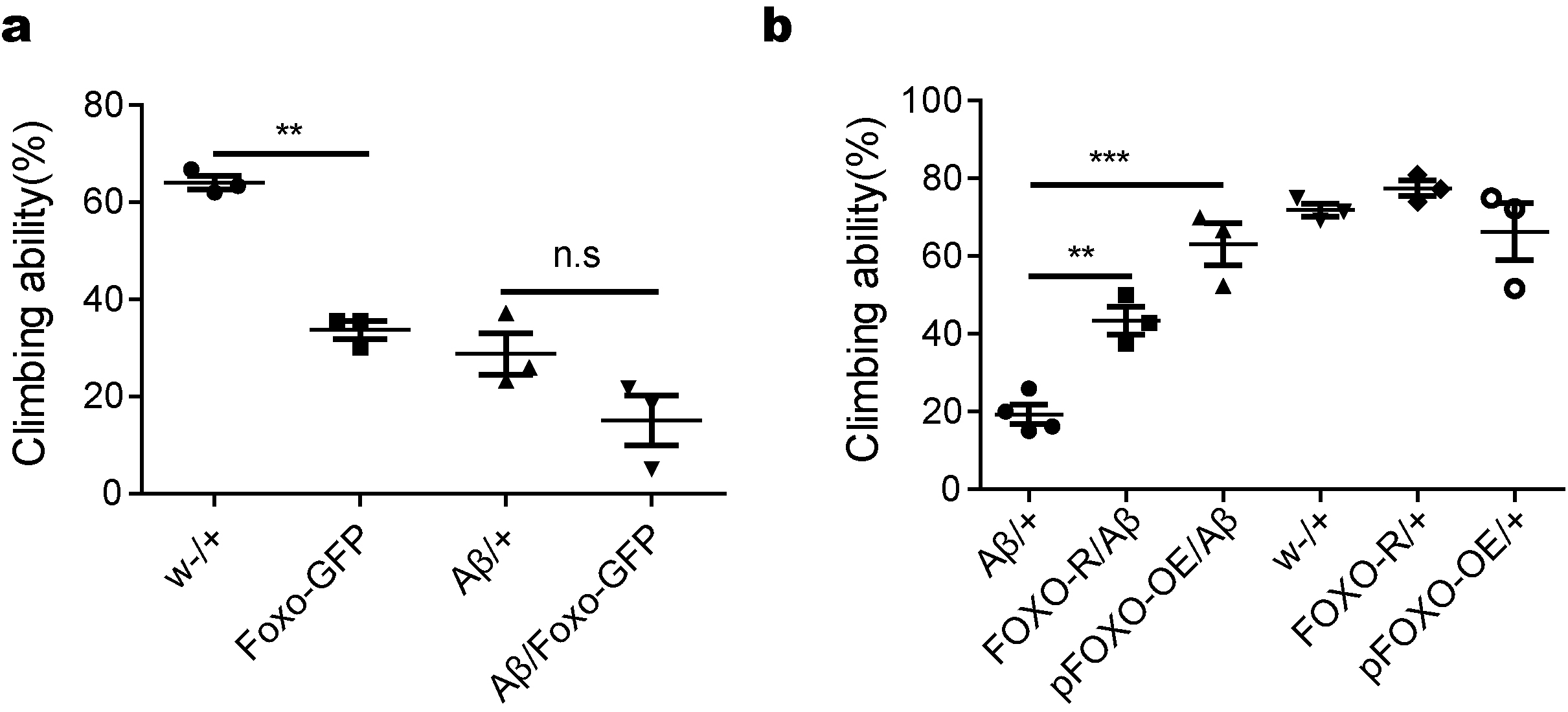
**

**Supplementary Figure 7. Effects of FOXO and S6K manipulation on the climbing ability of Aβ flies**

(**a**) Overexpression of FOXO-GFP could not rescue the climbing disability of Aβ flies. Data represent mean±SEM, **p<0.01. *Elav-Gal4* was used to drive Aβ and FOXO-GFP expression in fly CNS.

(**b**) Overexpression of *Drosophila* pFOXO and knock-down of *FOXO* improved the climbing ability of Aβ flies. Data represent mean±SEM, **p<0.01, ***p<0.001. *Elav-Gal4* was used to drive Aβ and OE or RNAi of genes in fly CNS. OE, overexpression.

**Supplementary Figure 8**


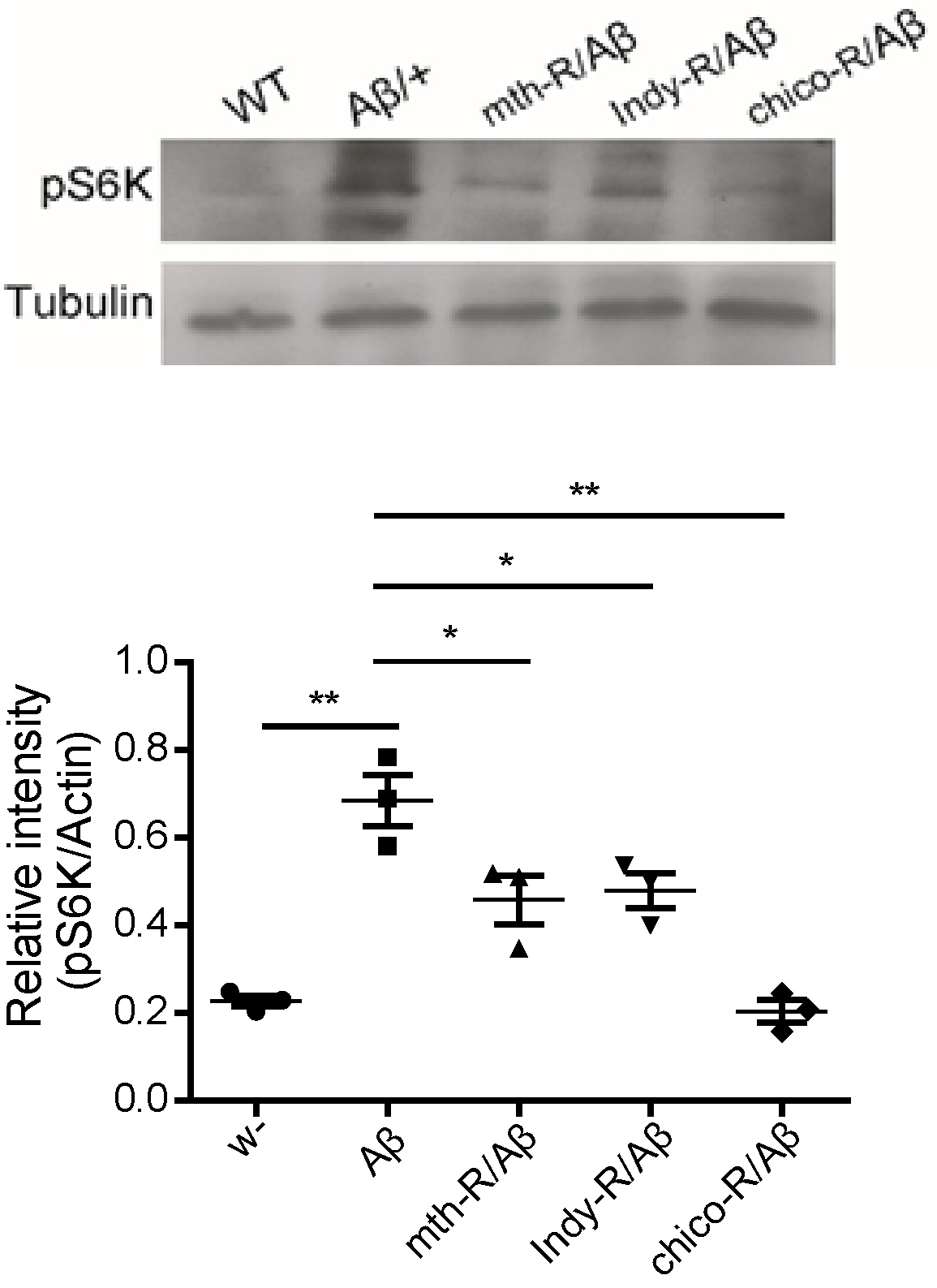


**Supplementary Figure 8. Down-regulation of fly *chico*, *mth* and *Indy* reduces phosphorylated S6K level in Aβ flies**

*Elav-Gal4* was used to express Aβ or knockdown *chico*, *mth* and Indy in the fly CNS; flies were raised at 29 ℃, and fly heads were harvested for Western blot analysis. Tubulin was used as the loading control. Results are reproducible in three independent Western blotting experiments, and only one is shown here. Data represent mean±SEM, *p<0.05, **: p<0.01.

**Supplementary Figure 9**

**
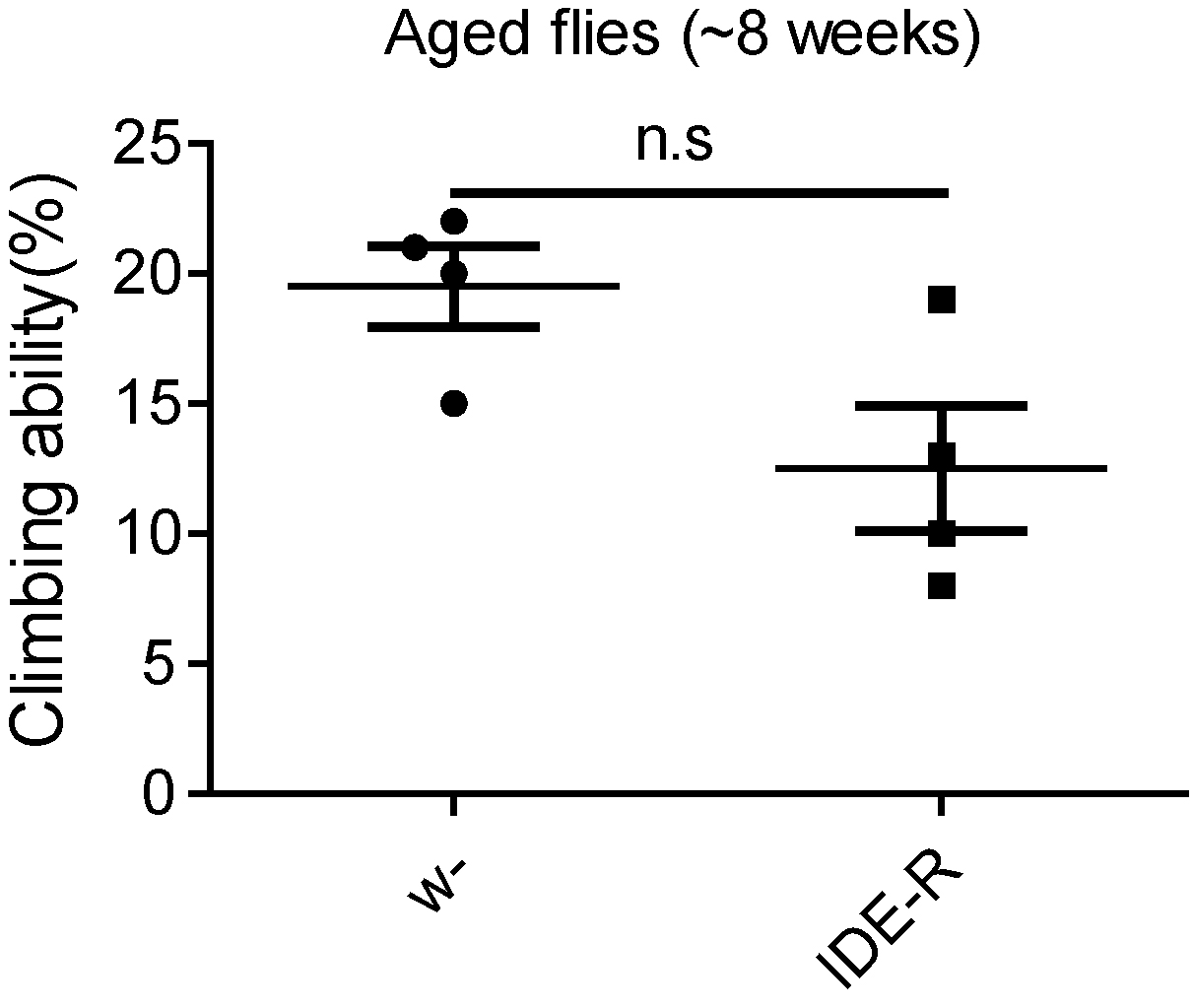
**

**Supplementary Figure 9. Effects of *IDE* RNAi on climbing ability of aged flies**

Down-regulation of fly *IDE* affects the climbing ability of aged flies. *Elav-Gal4* was used to knockdown IDE in the fly CNS; flies were raised at 25 ℃. Data represent mean±SEM, *p<0.05.

**Supplementary Figure 10**


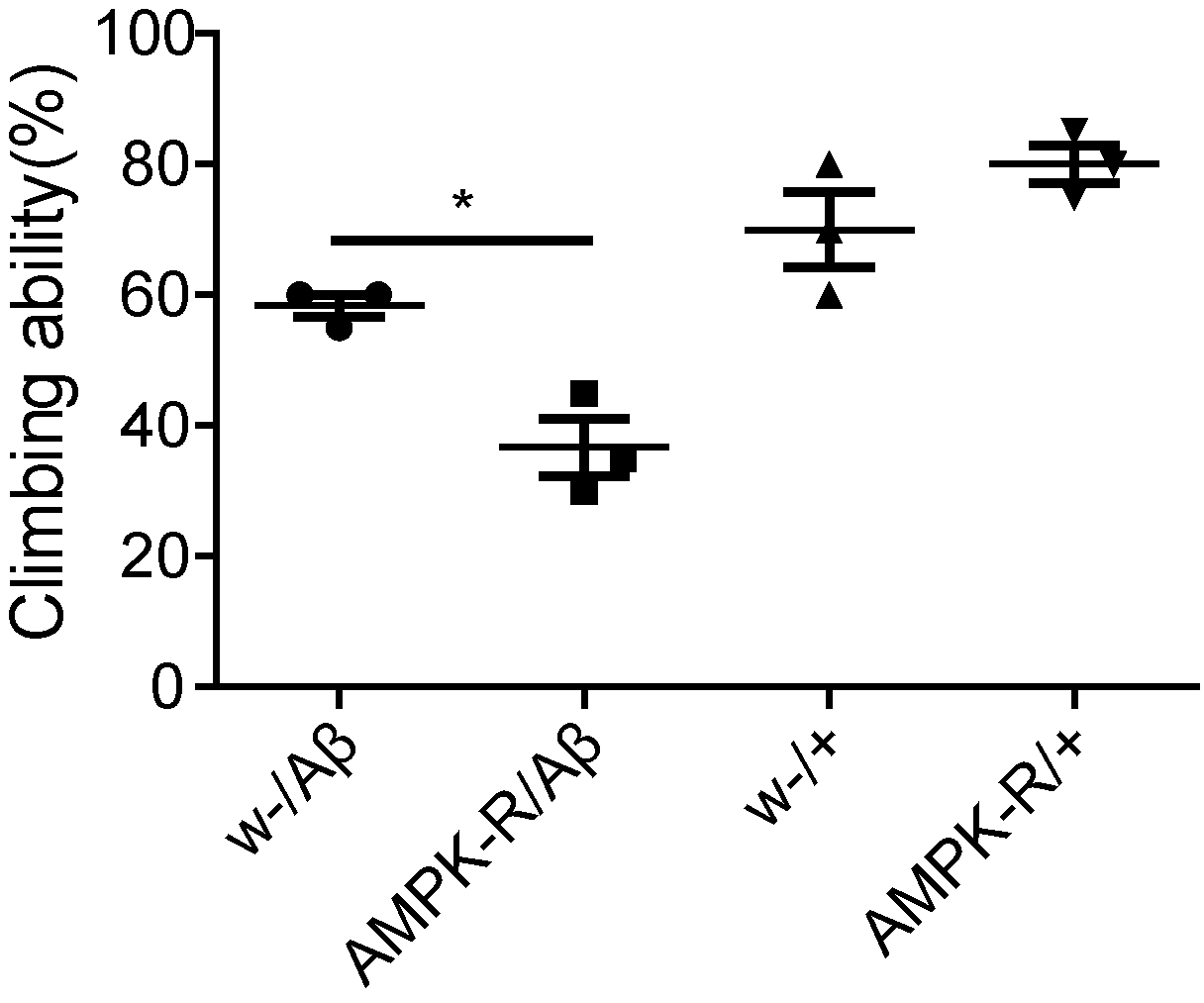


**Supplementary Figure 10. Effects of AMPK RNAi on climbing ability of Aβ flies**

Down-regulation of fly AMPK affects the climbing ability of Aβ flies. *Elav-Gal4* was used to express Aβ or knockdown of AMPK in the fly CNS; flies were raised at 29 ℃. Data represent mean±SEM, *p<0.05.

**Supplementary Figure 11**

**
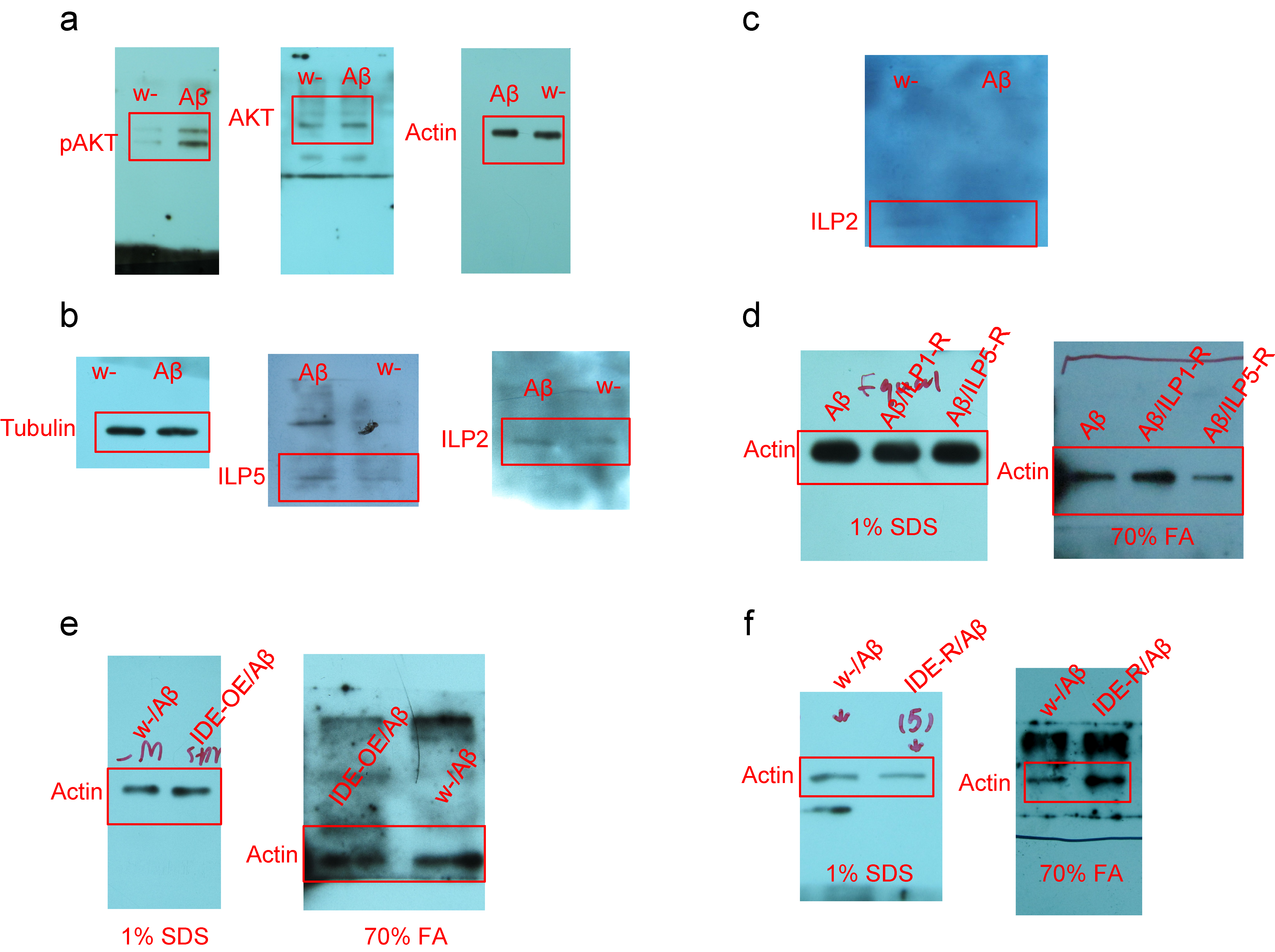
**

**Supplementary Figure 11. Uncropped Western blot images refer to main figure 3 and main figure 4.**

The names of the target proteins are indicated on each image. (**a**) refers to main figure **3b**, (**b**) refers to main figure **3c**, (**c**) refers to main figure **3e**, (**d**) refers to main figure **3f**, Actin Western blot, (**e**) refers to main figure **4a**, Actin Western blot, (**f**) refers to main figure **4b**, Actin Western blot.

**Supplementary Figure 12**


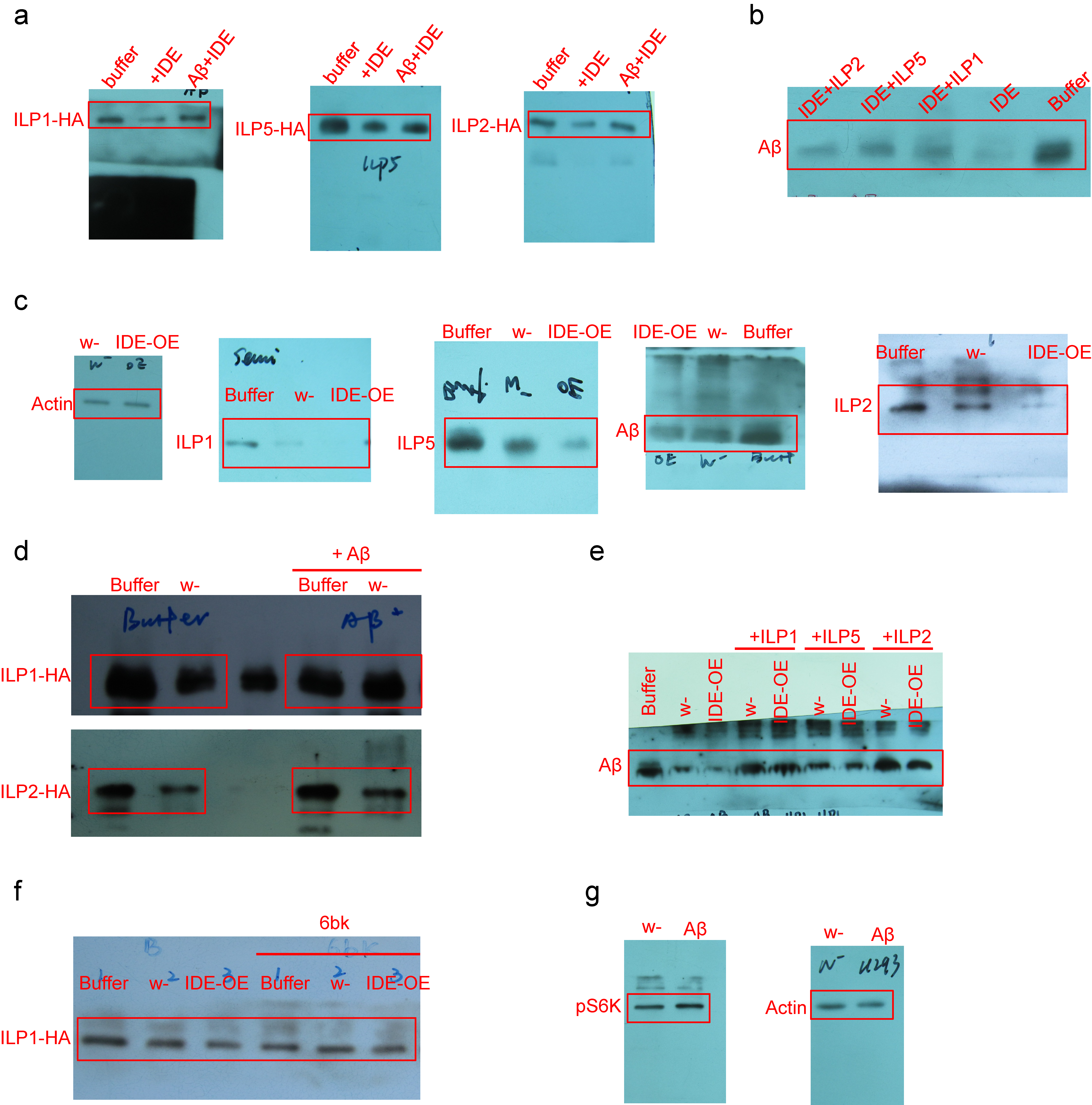


**Supplementary Figure 12. Uncropped Western blot images refer to main figure 5 and main figure 7.**

The names of the target proteins are indicated on each image. (**a**) refers to main figure **5a**, (**b**) refers to main figure **5b**, (**c**) refers to main figure **5c**, (**d**) refers to main figure **5d**, (**e**) refers to main figure **5e**, (**f**) refers to main figure **5f**, (**g**) refers to main figure **7b**.

**Supplementary Table 1. Genes tested in the genetic screening for Aβ suppressor**

| **Name** | **Gene #** | **Phenotype** |
| --- | --- | --- |
| ***chico*** | *CG5686* | RNAi rescued Aβ toxicity |
| ***mth*** | *CG6936* | RNAi rescued Aβ toxicity |
| ***Pcmt*** | *CG2152* | Without effect |
| ***Sir2*** | *CG5216* | Possibly without effect?  (RNAi affected climbing ability of control flies) |
| ***Cisd2*** | *CG1458* | Without effect |
| ***Hsf1*** | *CG5748* | Without effect |
| ***AMPK*** | \|  \| *CG3051* \| \| --- \| --- \| | RNAi enhanced Aβ toxicity |
| ***Indy*** | *CG3979* | RNAi rescued Aβ toxicity |

**Supplementary Table 2. Mass spectra analysis results of the purified ILP1-HA and ILP2-HA proteins.**

| **Accession** | **Description** | **Score** | **# Peptides** | **# PSMs** |
| --- | --- | --- | --- | --- |
| Q9VT51 | Probable insulin-like peptide 2 | 390.51 | 34 | 151 |
| Q9VT50 | Probable insulin-like peptide 1 | 400.79 | 60 | 179 |

**Supplementary Table 3. Primer sequences used in RT-PCR**

| rp49 forward | 5-GCACCAAGCACTTCATCC-3 |
| --- | --- |
| rp49 reverse | 5-CGATCTCGCCGCAGTAAA-3 |
| tobi forward | 5-TCTTTAAGCGCGCTGTGGATT-3 |
| tobi reverse | 5-AGGGTAGGGTATCCAAGGGAG-3 |
| Thor forward | 5-AGGAAGGTTGTCATCTCGGAT-3 |
| Thor reverse | 5-GAACTGTTCCTGGTCCTCAAT-3 |
| ILP2 forward | 5-GCCAGCTCCACAGTGAAGTT-3 |
| ILP2 reverse | 5-TCAGCCAGGGAATTGAGTAC-3 |
| S6K forward | 5-GACCTGGAGCTGCACGACCTG-3 |
| S6K reverse | 5-CCACCTTTGCCAAGGACCTTC-3 |
| IDE forward | 5-CACATGCTGTTCCTCGGCACC-3 |
| IDE reverse | 5-TGCTCAGAGTTTACCGCATTG-3 |
| ILP1 forward | 5-CTGTCCGATGCCATGGATGT-3 |
| ILP1 reverse | 5-TGTGGCGACGCATCTTGATC-3 |
| ILP5 forward | 5-CCCAGTTCTCCTGTTCCTGA-3 |
| ILP5 reverse | 5-GCCGAATGCTCGACAGTGAG-3 |
| Tor forward | 5-GACAGATATCAACGAGAAAA-3 |
| Tor reverse | 5-GTCAGGATGTGCTGGTAGAA-3 |
| Pi3K21B forward | 5-CGGACCTGTTCAATTC-3 |
| PI3K21B reverse | 5-ATTCTGAAGCAGCTGG-3 |
| chico reverse | 5-ATCCCAAGACACTTTG-3 |
| chico forward | 5-GCAAGTTGTCATTCAA-3 |
