# Supplementary material for: Insulin signaling in Drosophila melanogaster mediates Aβ toxicity
Source: Commun Biol. 2019 Jan 8;2:13. doi: 10.1038/s42003-018-0253-x (PMC6325060; doi:10.1038/s42003-018-0253-x)
Supplement: Supplementary file 3 — Description of Supplementary Data [file 42003_2018_253_MOESM3_ESM.docx]

**Description of Additional Supplementary Files**

**File Name**: Supplementary Data 1

**Description**: The source data underlying the graphs and charts presented in the main figures (excel file).
